# ROS Dependent Wnt/β-Catenin Pathway and Its Regulation on Defined Micro-Pillars—A Combined In Vitro and In Silico Study

**DOI:** 10.3390/cells9081784

**Published:** 2020-07-27

**Authors:** Susanne Staehlke, Fiete Haack, Anna-Christin Waldner, Dirk Koczan, Caroline Moerke, Petra Mueller, Adelinde M. Uhrmacher, J. Barbara Nebe

**Affiliations:** 1 Department of Cell Biology, Rostock University Medical Center, Schillingallee 69, 18057 Rostock, Germany; Anna-Christin.Waldner@med.uni-rostock.de (A.-C.W.); caroline.moerke@med.uni-rostock.de (C.M.); pemueller@hotmail.com (P.M.); barbara.nebe@med.uni-rostock.de (J.B.N.); 2 Modeling and Simulation Group, Institute for Visual and Analytic Computing, University of Rostock, Albert-Einstein-Str. 22, 18059 Rostock, Germany; fiete.haack@uni-rostock.de (F.H.); adelinde.uhrmacher@uni-rostock.de (A.M.U.); 3 Institute for Immunology, Core Facility for Microarray Analysis, Rostock University Medical Center, Schillingallee 70, 18057 Rostock, Germany; dirk.koczan@med.uni-rostock.de; 4 Department Science and Technology of Life, Light and Matter, University of Rostock, Albert-Einstein-Str. 25, 18059 Rostock, Germany

**Keywords:** human osteoblastic cells, micro-pillars, canonical Wnt pathway, β-catenin, microarray, gene profiling, reactive oxygen species (ROS), in silico, simulation

## Abstract

The physico-chemical surface design of implants influences the surrounding cells. Osteoblasts on sharp-edged micro-topographies revealed an impaired cell phenotype, function and Ca^2+^ mobilization. The influence of edges and ridges on the Wnt/β-catenin pathway in combination with the cells’ stress response has not been clear. Therefore, MG-63 osteoblasts were studied on defined titanium-coated micro-pillars (5 × 5 × 5 µm) in vitro and in silico. MG-63s on micro-pillars indicated an activated state of the Wnt/β-catenin pathway. The β-catenin protein accumulated in the cytosol and translocated into the nucleus. Gene profiling indicated an antagonism mechanism of the transcriptional activity of β-catenin due to an increased expression of inhibitors like ICAT (inhibitor of β-catenin and transcription factor-4). Cells on pillars produced a significant reactive oxygen species (ROS) amount after 1 and 24 h. In silico analyses provided a detailed view on how transcriptional activity of Wnt signaling is coordinated in response to the oxidative stress induced by the micro-topography. Based on a coordinated expression of regulatory elements of the Wnt/β-catenin pathway, MG-63s are able to cope with an increased accumulation of β-catenin on micro-pillars and suppress an unintended target gene expression. Further, β-catenin may be diverted into other signaling pathways to support defense mechanisms against ROS.

## 1. Introduction

Biofunctional materials which replace damaged tissue and stimulate cell regeneration are currently in great demand and will be in the future. Titanium (Ti) and titanium-based alloys are the most common metallic materials in implant applications due to their good biocompatibility, mechanical compliance, and high corrosion resistance [1,2,3]. The rapid cellular acceptance in the tissue can be optimized by physico-chemical surface functionalization, e.g., topographical cues [4,5,6]. Via adhesion receptors, cells are able to perceive the material surface properties and also the extra cellular matrix (ECM) deposited at the interface [7]. Initial cell adhesion [8] and downstream intracellular signaling cascades [9] finally determine the cell physiology and cell fate [10,11]. However, the underlying mechanisms are not fully understood. 

The previous systematic analyses revealed that a defined topographical environment with cubic pillars reduced cell functions, such as proliferation and ECM-synthesis (collagen type I and fibronectin) [9,12,13]. Phagocytosis-like processes occurred in MG-63 osteoblasts when the topography featured sharp corners and edges; these were accompanied by a reorganization of the actin cytoskeleton into short fragments around and on top of the pillars (Figure S1) [9,12,13]. In addition, a reduced capacity of the osteoblasts to mobilize the “second messenger” calcium ions (Ca^2+^) was observed [9]. These studies have aroused interest in additional research on the role of mechanotransduction of external signals. 

The Wnt signaling pathways are known to be one of the most important signal transduction systems in tissue homeostasis [14,15,16] and play a crucial role in bone regeneration, bone formation, and osseointegration. It is known that surface topographical features can activate the most important pathway—the canonical Wnt or Wnt/β-catenin pathway [11,17,18,19,20,21]. 

### Wnt/β-Catenin Pathway

**Scheme 1:**
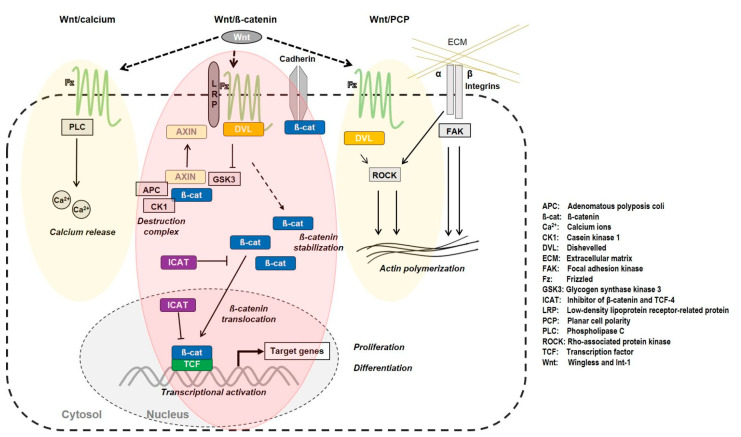
Scheme of the molecular basis of the Wnt pathway. A central role in indirect mechanotransduction is played by Wnt signaling. In the inactive state, the key component β-catenin (β-cat) is constantly degraded by the destruction complex consisting of AXIN, adenomatous polyposis coli (APC), glycogen synthase kinase 3 (GSK3) and casein kinase 1 (CK1). In the active state, initiated by Wnt-binding to the receptor complex Frizzled (Fz) and Low-density lipoprotein receptor-related protein (LRP), the recruitment of Dishevelled (DVL) occurs. This further recruits components of the destruction complex, such as AXIN. Subsequently, β-catenin accumulates in the cytosol and translocates into the nucleus where it acts as a co-activator of transcription factors (TCF). β-catenin is regulated by the inhibitor of β-catenin and TCF-4 (ICAT).

The key component of the canonical Wnt pathway is the protein β-catenin [16]. In the inactive state, β-catenin is constantly degraded by the destruction complex which consists of the four components AXIN, adenomatous polyposis coli (APC), glycogen synthase kinase 3β (GSK3β) and casein kinase 1 (CK1) (Scheme 1) [16]. The activated state of the Wnt/β-catenin pathway is initiated by the binding of the glycoprotein Wnt to a receptor complex, which comprises the Frizzled (Fz) receptor and the low-density lipoprotein receptor-related protein (LRP) [22]. Wnt ligands are synthesized in the endoplasmic reticulum and extracellularly secreted by binding to the transmembrane protein Wntless [16,23]. When the ligand Wnt binds to its receptor complex Fz-LRP [22], a series of recruitment and phosphorylation events take place (Scheme 1) [14]. The resulting signalosome leads to a disruption of the destruction complex, partly due to the inhibition of AXIN [24]. As a result, cytoplasmic β-catenin levels are stabilized and accumulated in the cytosol [14,18]. This triggers the translocation of β-catenin into the nucleus where it acts as co-activator for T-cell factor/lymphoid enhancer factor (TcF/LEF) transcriptional factors (TCF), and finally drives the specific expression of target genes [19] to regulate various physiological processes (Scheme 1) [15]. Different cellular strategies exist to control β-catenin and the TCF/β-catenin transcriptional activation, e.g., via an inhibitor of β-catenin and TCF-4 (ICAT) [23,25,26,27]. Furthermore, the regulation of β-catenin accumulation can be achieved by the production of reactive oxygen species (ROS) in cellular differentiation processes [28].

Based on the available data, topography-induced osteogenic differentiation appears to be associated with an activated Wnt/β-catenin pathway [11,17,18]. But topographies with sharp edges impaired the cell differentiation [12,13]. Therefore, the question arises whether and how the Wnt/β-catenin pathway in osteoblasts is affected by a defined surface micro-topography.

The aim of this study was to understand the effects of sharp-edged geometrical micro-pillars on components of the Wnt/β-catenin pathway in human MG-63 osteoblasts. We hypothesize that a defined surface micro-topography modify the signal transduction and ultimately prevent the activation of context-specific target genes. The expression of regulator of Wnt signaling: β-catenin, inhibitors like ICAT, SOX17 (SRY-box transcription factor 17) and transcription factors (TCF4) was quantified in vitro and simulated in silico. The influence of cellular oxidative stress was evaluated as additional trigger of β-catenin. Finally, the complex interplay of ROS, β-catenin and specifically expressed inhibitors and transcription factors was evaluated in silico. The model illuminates the redox-sensitive pathway that activates β-catenin in a receptor and Wnt-independent manner as well as the corresponding cellular and transcriptional response mechanisms that suppress (unintentional) Wnt/β-catenin target gene expression [28,29].

## 2. Materials and Methods

### 2.1. Titanium Samples—Micro-Pillars

Silicon wafers with a size of 1 cm² were sputter-coated with 100 nm titanium (Ti, pure > 99.999%). The unstructured bulk material served as reference (Ref). For the defined micro-pillars, the silicon wafers were structured by deep reactive-ion etching (DRIE) (Center for Microtechnologies, ZFM, University of Technology Chemnitz, Chemnitz, Germany). The resulting geometrical sharp-edged pillars had the dimensions 5 × 5 × 5 µm (length × width × depth) (P5) [9,13,30]. The Ti-samples were sterilized with 70% ethanol (Walter-CMP GmbH & Co. KG, Kiel, Germany) for 15 min and rinsed with phosphate-buffered salt solution (PBS, PAA Laboratories, Pasching, Australia).

### 2.2. Cell Culture and Subcellular Fractionation

Osteoblast-like human cells MG-63 (American Type Culture Collection, ATCC^®^ CRL-1427^™^), Manassas, VA, USA) were cultured in Dulbecco’s modified Eagle’s medium (DMEM, Life technologies GmbH, Darmstadt, Germany) with 10% standardized fetal calf serum (FCS, Biochrom FBS Superior, EU-approved, Merck KgaG, Darmstadt, Germany) and 1% gentamycin (Ratiopharm GmbH, Ulm, Germany) (complete media) at 37 °C in 5% CO_2_ according to the protocols described previously [9,13,31]. The experiments were carried out at a cell confluence of ~70%. The MG-63s were dissociated by the addition of Trypsin-EDTA (ethylenediaminetetraacetic acid, 0.05%; PAA), seeded onto the Ti-samples and cultivated for up 96 h.

To analyze the protein content in subcellular fractions, 5 × 10^4^ cells were cultivated on the samples within 24 h and prepared using the Subcellular Proteome Extraction Kit (Calbiochem^®^, Merck KgaG). In brief, protein degradation was prevented by a protease inhibitor cocktail and Benzonase^®^ nuclease was used to remove contaminating nucleic acids. Then MG-63s were incubated with the respective extraction buffer to get the cytosolic-, membrane/organelle-, and nucleic protein extract fraction. Proof of subcellular fractionation were carried out by SDS-Page (Sodium Dodecyl Sulfate PolyAcrylamide Gel Electrophoresis) and assign selected marker proteins were detected using immunoblot (see Section 2.6) (Figure S2).

### 2.3. Gene Expression Profiling

The human Clariom^™^ S Array (Affymetrix^®^ Inc., St. Clara, CA, USA) were performed for mRNA (messenger ribonucleic acid) expression profile. After 24 h of cultivation (5 × 10^4^ cells) on the surfaces, RNA extraction was done with the RNeasy Kit (Qiagen, Hilden, Germany) according to the manufacturer’s protocol. The total RNA samples were quantified spectrophotometrically (NanoDrop 1000, Thermo Fisher Scientific, Inc., Waltham, MA, USA) and the integrity was controlled using the Agilent Bioanalyzer 2100 with the RNA Pico chip kit (both from Agilent Technologies, St. Clara, CA, USA). RIN (RNA integrity number) values between 9.4 and 10 were achieved. A 100 ng whole RNA sample was used as the starting material in the GeneChipR Whole Transcript Sense Target Labeling protocol (Affymetrix). The microarray hybridization was done using the Affymetrix Clariom^™^ S Array Kit according to the manufacturer’s instructions (detailed described [32]). 

The mRNA expression profiling and analysis of raw data was performed using the Transcriptome Analysis Console (TAC) version 4.0.2.1.5 (Applied Biosystems, Thermo Fisher Scientific Inc., Santa Clara, CA, USA). The subsequent evaluation of the datasets P5 versus Ref (triplicates from independent experiments) was summarized by Signal Space Transformation-Robust Multi-Chip Analysis (SST-RMA). Technical replicate groups were summarized by linear models for microarray data (LIMMA, eBayes) statistics. Differentially expressed genes (DEG) were identified using filter parameters, the SST-RMA Algorithm with the settings fold change > 1.5 or <−1.5, LIMMA *p*-value < 0.05. Furthermore, TAC links directly to multiple public databases like WikiPathways to identify the molecular events associated with the Wnt signaling. A table containing an overview of genes related to Wnt signaling can be found as part of the supplementary material (Table S1).

### 2.4. Immunofluorescence and Image Analysis

MG-63 osteoblasts (4 × 10^4^) were seeded on each sample for 24 h. Cells were fixed with 4% paraformaldehyde (PFA; Sigma-Aldrich, St Louis, MO, USA) for 10 min at room temperature (RT) followed by permeabilization with 0.1% Triton X-100 (Merck KgaG). MG-63s were incubated with the primary antibodies: β-catenin (CTNNB; Acris, OriGene Technologies Inc., Rockville, MD, USA; 1:100), inhibitor of β-catenin and TCF-4 (ICAT, 5C6; Santa Cruz Biotechnology, Inc., Dallas, TX, USA; 1:100) and SOX17 (Clone ID: OTI3B10; Acris; 1:50) at RT for 1 h and then fluorescence-conjugated secondary antibodies (dilution 1:200): Alexa Fluor^®^ 488-labeled goat anti-rabbit or -mouse (Life Technologies), Goat F(ab’)2 anti-mouse IgG-Cy3 (Indocarbocyanin; dianova GmbH, Hamburg, Germany) for 30 min at RT. Finally, the samples were fixed on microscope slides and mounted using Fluoroshield™ with DAPI (4′,6-diamidino-2-phenylindole; 1.5 µg/mL, Sigma-Aldrich). The location and expression of proteins were visualized by the inverted confocal laser scanning microscope LSM 780 (63 × Plan-Neofluar, 1.25 oil/0.17, Carl Zeiss AG, Oberkochen, Germany). 

The software ZEN 2011 (ZEISS Efficient Navigation, ZEN 2011 SP4, black or blue edition, Carl Zeiss) was used for image analysis. For MG-63s on micro-pillars, several images were acquired in the “Z-stack” function (3–10 slices with an interval of 1 µm, pinhole 0.7 µm) and then generated a 3D-reconstruction (overlay). Confocal microscopy with 3D Z-stack generation was performed to visualize the protein localization on top of and between the micro-pillars to understand protein localization that was not limited to a single dimension. Using the “Profile” function, intensity profiles of the individual fluorescence’s of certain regions in the image can be created.

### 2.5. Flow Cytometry

The MG-63s (4 × 10^4^) cultivated for 1 h or 24 h on the Ti-samples were harvested by detachment with Trypsin-EDTA, fixed with 4% PFA (10 min) and permeabilized with 0.1% Triton X-100 (10 min). The cells were resuspended in the primary antibodies (dilution 1:50): β-catenin (CTNNB; Acris) and SOX17 (Acris) for 30 min each, followed by the secondary antibodies (dilution 1:200): Alexa Fluor^®^ 488-labeled goat anti-rabbit or -mouse (Life technologies). After staining, the suspended cells were analyzed using a BD FACSCalibur^™^ (BD Biosciences, Franklin Lakes, NJ, USA). The fluorescence intensity of at least 10,000 events was measured using CellQuest Pro 4.0.1 (BD Biosciences) and calculated as the FL-1 mean channel by FlowJo_V.10.1r1 (FlowJo, LLC; BD Becton Dickinson and Company, Franklin Lakes, NJ, USA).

### 2.6. Quantitative Western Blot Analysis

Immunoblots were done from total lysates (osteogenic marker, 96 h) and subcellular fraction lysates (β-catenin, 24 h) of MG-63s on the Ti-samples. The protein content was measured using a Qubit^®^ protein assay kit (Thermo Fisher Scientific Inc.). The cellular protein (25 µg per lysate) were loaded into Criterion^™^TGX Stain-Free^™^-gel (4–15%; Bio-Rad Laboratories, Hercules, CA, USA) and separated using SDS-PAGE (Bio-Rad). Afterwards, the proteins were transferred onto PVDF membrane (polyvinylidene fluoride; Roche, Mannheim, Germany) by electro blotting (150 mA overnight). The PVDF membranes were blocked with 5% low fat milk solution (Roth, Karlsruhe, Germany) for 1 h, and incubated with the primary antibodies: β-catenin (Acris; rabbit polyclonal 1:1000); Apg1 (120 D-12; Heat Shock Protein Family A; Santa Cruz: mouse monoclonal 1:1000), pan-cadherin (E-11; Santa Cruz; mouse monoclonal 1:1000); TCF-1 (T cell factor-1; C-5; Santa Cruz; mouse monoclonal 1:1000) bone sialo protein 2 (BSP-2; rabbit polyclonal; 1:500), collagen type I (Col1; rabbit polyclonal; 1:3000); fibronectin (FN; rabbit monoclonal; 1:1000) and GAPDH (glyceralaldehyde 3-phosphate dehydrogenase, A-3; Santa Cruz; mouse monoclonal 1:1000) at 4 °C overnight. As secondary antibodies: horseradish peroxidase (HRP)-conjugated monoclonal anti rabbit or mouse IgG (Dako Denmark A/S, Glostrup, Denmark; 1:10,000) was used at RT for 1 h. Protein expression was detected using enhanced chemiluminescence-substrate (Thermo Fisher Scientific Inc.). The band intensity was quantified by densitometry in the ChemiDoc^™^ MP imager (Bio-Rad) using the software Quantity One^®^ and Image Lab^™^ (Bio-Rad). In order to control the equal protein amount or confirm the specificity of the purity of the fractions markers for nucleus, cytosol and membrane, Stain-Free-gel or PVDF membrane were visualized (Figure S2).

### 2.7. Bio-Plex Luminex Bead Multiplex Analysis

The secreted proteins osteocalcin (OCN) and osteopontin (OPN) from lysates of the cell culture supernatant after 96 h were analyzed using MILLIPLEX MAP Kit for Human Bone Magnetic Bead Panel (Merck KGAA) according to the manufacturer’s recommendations. Briefly, the bead mix was transferred to each well of the 96-well plate and mixed with lysates over night at 4 °C shaking with 300 rpm. Subsequently, incubation was performed with a detection antibody solution (30 min at RT, 300 rpm) and afterwards with a Streptavidin-Phycoerythrin solution (30 min at RT, 300 rpm). The protein quantification, mean fluorescence intensity (MFI), was measured with the Bio-Plex^®^200 System (Bio-Rad) and the Bio-Plex Manager^™^ 4.1.1 software (Bio-Rad). The MFI values were normalized to the protein concentration determined by Bradford method.

### 2.8. Enzyme-Linked Immunosorbent Assay (ELISA)

For quantitative protein detection of ICAT in the cell fraction lysates of MG-63s within 24 h, the ICAT ELISA kit (human beta catenin interacting protein 1 (CTNNBIP1); MyBioSource, Inc., San Diego, CA, USA) was used according to the manufacturer’s instructions. Briefly, the 96-well pre-coated microtiter plate was loaded with equal amounts of the protein as well as the standards. Lysates were labeled with CTNNBIP1-HRP conjugates for 1 h and subsequently incubated with the substrate for HRP enzyme at 37 °C for 20 min. Immediately after stopping the reaction, the optical density (O.D., intensity of the color) could be measured using Anthos Reader 2010 (Anthos Mikrosysteme GmbH, Friesoythe, Germany) at 450 nm. For the calculation of ICAT protein expression level, a standard curve was plotted relating the O.D. to the standard concentration.

### 2.9. Detection of Reactive Oxygen Species (ROS) and Lipid Peroxidation

The production of intracellular ROS were detected using DCF-DA Cellular ROS detection assay kit (Abcam, Cambridge, UK). Therefore, a trypsinated MG-63 cell pellet was resuspended in a dye solution consisting of 2′,7′-dichlorofluorescein diacetate (DCF-DA, 20 µM) and then shaken (300 rpmi) at 37 °C for 30 min in the dark. The stained MG-63s were resuspended in complete DMEM (without phenol red; Life technologies GmbH). Afterwards, 1 × 10^5^ osteoblasts were cultivated on the samples within 24 h. In the presence of an ROS, the DCF-DA is oxidized to the fluorescence compound DCF [13]. The CellROX reagents (Thermo Fisher Scientific) were used to evaluate ROS primarily in the nucleus and mitochondria (CellROX^®^Green) or cytosol (CellROX^®^Orange) according to the manufacturer’s instructions. After 24 h adherence on the samples, cells were washed with PBS and loaded with 5 µM of one fluorogenic probe at 37 °C for 30 min. 

Lipid peroxidation of 24 h adherent MG-63s was analyzed using the Image-iT^®^ Lipid Peroxidation Kit (C11-BODIPY, Thermo Fisher Scientific). C11-BODIPY (581/591) fluorescence was measured using a filters for FITC and TexasRed. Upon oxidation (ROS production), fluorescence changes from red to green and its ratio indicates the degree of lipid peroxidation. As a positive control, cells were treated with 0.5% hydrogen peroxide (H_2_O_2_) or cumene hydroperoxide (CH) (Figure S3). The fluorescence intensity was measured by a fluorescence microplate reader (Tecan infinite M200; Tecan i-control, 1.9.17.0) or observed under the LSM 780 by 40× C Apochromat (1.2 W Korr M27) objective (Carl Zeiss) with additional nucleus staining (Hoechst 33342; Thermo Fisher Scientific).

### 2.10. Statistics

All in vitro experiments were repeated at least three times with independently passaged cells. To evaluate statistical differences and to prepare the graphs, the GraphPad Prism 7 software for Windows (GraphPad Software Inc., La Jolla, CA, USA) was used. Data were normalized either to unstructured bulk material (Ref) or time point (1 h). After determining the normal distribution by Kolmogorov-Smirnov, subsequent statistical test was done: the Mann-Whitney U test (protein expression by FACS; osteogenic marker analysis), paired *t*-test (ROS and lipid peroxidation detection) or the multiple *t*-test (β-catenin, ICAT in subcellular fractions). Herein, a probability value of *p* < 0.05 was accepted as indicating a significant difference. Data were expressed as mean ± s.e.m. (standard error of the mean).

### 2.11. In Silico Experiments: Modeling the Wnt/β-Catenin Pathway in Osteoblasts 

The simulation model is defined in ML-Rules, a multi-level, rule-based modeling language [33]. ML-Rules allows the specification and simulation of complex systems biology models at multiple levels of organization. The semantics of ML-Rules is based on continuous-time Markov chains (CTMC) [34]. For all simulation experiments performed in this study, a stochastic simulator that is based on the Stochastic Simulation Algorithm (SSA) was used [34]. For the specification and execution of the model SESSL (The Simulation Experiment Specification on a Scala Layer) [35] was used. Based on the bindings between SESSL and ML-Rules [36], various simulation experiments have been executed. The specifications of these experiments in SESSL, the model in ML-Rules and the R-Script to replicate the figures shown in the paper are available in the GitHub repository https://github.com/SFB-ELAINE/SI_Staehlke_MDPI_Cells. A table containing all parameter values employed in the (fitted) model as well as the model implementation in ML-Rules can be found as part of the supplementary material (Table S2 and Figure S4). 

## 3. Results

### 3.1. Topography-Dependent β-Catenin Accumulation and Translocation into the Nucleus 

The stabilization, accumulation, and translocation of the β-catenin into the nucleus is a marker for the activation of the Wnt/β-catenin pathway [15,16,23] and was examined by flow cytometry, laser scanning microscopy (LSM) and Western Blot (subcellular fractionation) within 24 h MG-63 cell cultivation on defined sharp-edged micro-pillars (P5).

**Figure 1:**
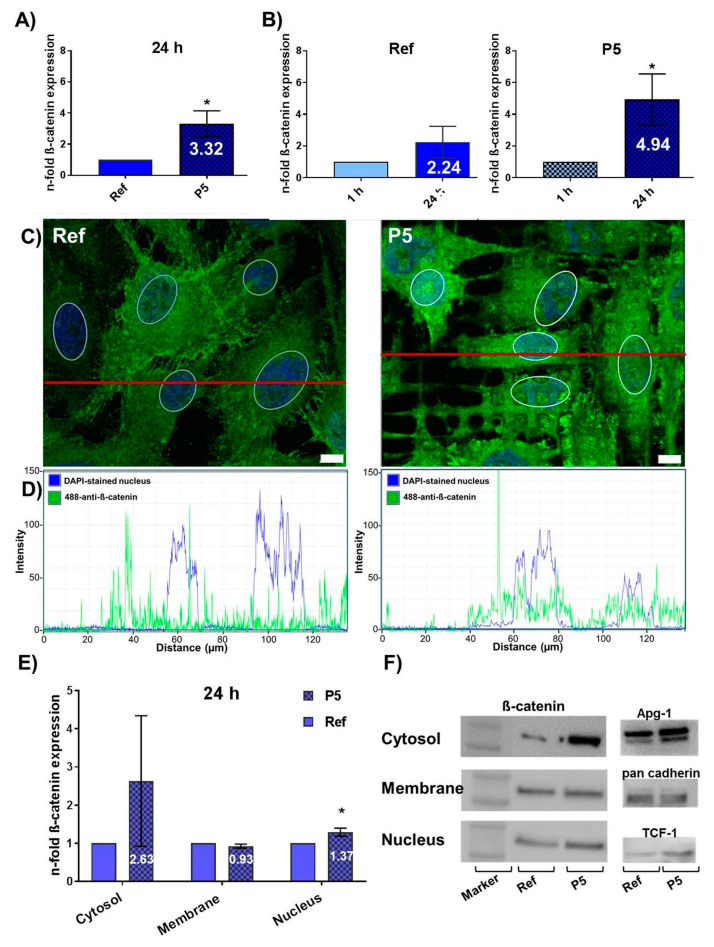
The β-catenin protein expression and localization in MG-63 osteoblasts on micro-pillars (P5). (**A**) Flow cytometric analysis of β-catenin total protein level in MG-63s after 24 h. Note that β-catenin was significantly increased in MG-63s on P5. (FACSCalibur, BD Biosciences; Ref values normalized to 1; mean ± s.e.m. of 4 independent experiments; Mann-Whitney U test; * *p* < 0.05). (**B**) Flow cytometric analysis of the time dependent β-catenin total protein expression after 1 h and 24 h on Ref and P5. Note that the expression of β-catenin on P5 was significantly increased after 24 h compared to 1 h, which indicated the accumulation of the protein. (FACSCalibur, BD Biosciences; 1 h values normalized to 1; mean ± s.e.m. of 4 independent experiments; Mann-Whitney U test; * *p* < 0.05). (**C**) Immunofluorescence of β-catenin in MG-63s on P5 compared with unstructured Ref after 24 h (green: Alexa Fluor^®^ 488-anti-β-catenin, blue: nucleus, DAPI (4′,6-diamidino-2-phenylindole); LSM 780, Carl Zeiss; magnification 63×, P5 immunofluorescent image is 3D confocal z-stack overlay, scale bar: 10 µm). Note that on Ref, the MG-63s showed a homogeneous distribution of β-catenin in the cell. On P5, β-catenin was mainly distributed in the cytosol and translocation in the nucleus was visible. (**D**) Profile of fluorescence intensity recorded at specific region across the fluorescence images (red line, 1C). Note on P5 the β-catenin profile (green) is detectable in the cytosol as well as in the nucleus (blue) with the same intensity. In contrast, on Ref the intensity of β-catenin (green) can rarely be detected in the core regions (blue). (Zeiss, ZEN function “Profile”; Pinhole section: 0.7 µm). (**E**) Immunoblotting of cell lysates of subcellular fractions and detection of β-catenin (24 h). Note that MG-63s on P5 indicated a trend to higher expression of β-catenin in the cytosol, and a significantly increased expression in the nucleus. (Ref values normalized to 1; mean ± s.e.m., 4 independent experiments; multiple *t*-test; * *p* < 0.05). (**F**) Representative Western blot bands of the β-catenin level (92 kDa) in MG-63s fractions on P5 compared to Ref (one out of four experiments, marker: upper line = 100 kDa; lower line: 75 kDa). On the right side, the selected marker proteins for the corresponding subcellular fraction are presented: cytosol—Apg1 (Heat Shock Protein Family A), membrane—pan-cadherin and nucleus—TCF-1 (T cell factor1).

In flow cytometry a significantly increase in total protein expression of β-catenin in cells on P5 compared with Ref after 24 h was observed (Figure 1A). Additionally, the total β-catenin protein expression increased over time in cells on P5 from 1 h to 24 h, but not on Ref (Figure 1B).

On Ref β-catenin appears evenly distributed throughout the cytosol with an increased localization along the cell membrane in cell-cell contacts. On P5 a general higher occurrence was detected and β-catenin was distributed mainly in the cytosol (Figure 1C). Also, translocation of β-catenin into the nucleus was observed on P5 (Figure 1D).

Also the quantification of β-catenin protein level in MG-63 subcellular fractions using Western Blot revealed a slightly higher level in the cytosol as well as a significantly increased expression in the nucleus on P5 versus Ref. No topographical influence could be observed in the protein expression of β-catenin in the membrane fraction (Figure 1E,F).

Taken together, MG-63 cells on P5 indicated an activated state of the Wnt/β-catenin pathway due to the accumulation of the key effector β-catenin in the cytosol and their transduction in the nucleus after 24 h.

### 3.2. Gene Regulation of Wnt Signaling Components Dependent on Micro-Pillars 

**Figure 2:**
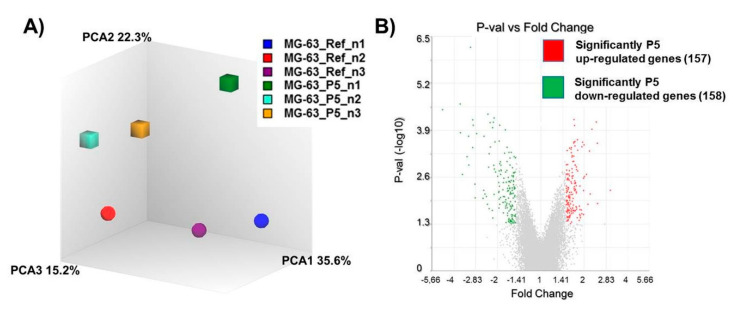
Gene expression profiling of MG-63 cells on micro-pillars (P5) compared to unstructured reference (Ref). (**A**) Principal component analysis (PCA) 3D mapping from complex microarray datasets of MG-63s on P5 (upper row, square) and on Ref (lower row, circle) to determine the variation of gene profiles (21,448 genes) of the individual expression profiles of the 3 independent experiments. Fractions of percentage of variation out of the total mapped difference (73.1%) among samples were signified on each axis. Note that the gene expression profile of MG-63s on P5 is clustered differently in each of the corresponding experimental approaches compared to the cells on Ref (vertical shift). (**B**) Volcano Plot visualization of differentially regulated genes in cells on P5 compared with Ref (red scatter plot = P5 up regulated genes; green scatter plot = P5 down regulated genes). Note that the colored points indicated a significantly different regulation in the mRNA expression pattern on P5 versus Ref with algorithm setting. Only 315 genes (1.47%) pass the filter criteria: Fold change > 1.5 or <−1.5, LIMMA (linear models for microarray data) statistics *p* < 0.05. (Analysis by TAC 4.0 software (Transcriptome Analysis Console)).

In order to obtain information about the overall composition of the analyzed complex microarray datasets and to determine the direction of variability, the principal component analysis (PCA) was performed (Figure 2A; PCA1-3 of 73.1%). The approach showed differences between the respective group gene expression profiles (Figure 2A). This indicates that the group profiles are divergent and separately clustered, which allowed for analysis of the differences in gene regulation (Figure 2A). In addition, similarities of the individual values within a group were shown, indicating proximity within the profile. 

The signal space transformation robust multi-array average (SST-RMA) algorithm identified expression values for 21,448 annotated gene clusters, which were further filtered with the criteria fold change cutoff, fold change > 1.5 or <−1.5, linear models for microarray data (LIMMA) *p* < 0.05, and for the identification of differentially regulated genes (Figure 2B). Of these, 315 (1.47%) genes were detected and marked as differently regulated in osteoblasts on P5 compared to Ref under the criteria fold change cutoff. In summary, 157 genes (49.8%) were up-regulated due to P5, while 158 genes (50.2%) were down-regulated due to P5 compared to Ref (Figure 2B). Under the filter criteria, 6 genes have been identified that were significantly (*p* < 0.05) differently regulated regarding the Wnt/β-catenin pathway (Table 1). 

**Figure 3:**
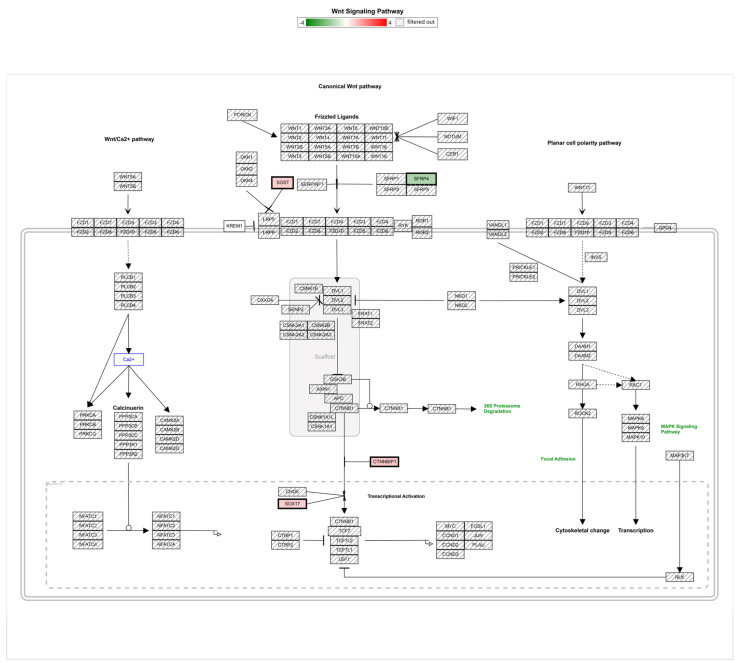
Illustration of the analyzed Wnt signaling genes in MG-63s on micro-pillars P5 compared with unstructured Ref. Note in particular the regulation of the Wnt/β-catenin pathway: P5 up-regulated genes (red-backed)—sclerostin (*SOST*), inhibitor of β-catenin and TCF-4 (ICAT), SRY-box transcription factor 17 (*SOX17*); P5 down-regulated genes (green-backed)—secreted frizzled-related protein 4 (*SFRP4*). In contrast, the non-canonical Wnt signaling pathways (Wnt/calcium and Wnt/planar cell polarity (PCP)) showed no difference in mRNA (messenger ribonucleic acid) expression (filtered out). Illustration of the Wnt-WikiPathways. (TAC 4.0.2.1.5. software (Transcriptome Analysis Console)).

The datasets were evaluated according to the pathways tool WikiPathways. Figure 3 summarizes these representations of Wnt pathways. Both non-canonical Wnt pathways—Wnt/calcium and Wnt/planar cell polarity (PCP)—indicated no altered mRNA expression profile. Regulated mRNA profiles were apparent solely in the Wnt/β-catenin pathway (Table 1). Interestingly, the mRNA expression of β-catenin remained unchanged (fold change 1.08). Further Wnt/β-catenin pathway components such as ligands Wnt, receptors like Frizzled (Fz) or low-density lipoprotein receptor-related protein (LRP) and Dishevelled (DVL) revealed no significant regulation in gene expression due to the underlying micro-topography. Also, target genes such as *AXIN2* or osteoblast-specific ones were not influenced on the mRNA expression level (Table S1). All in all, 5 out of 6 significantly expressed components were upregulated in cells on P5. The activated state of the Wnt/β-catenin pathway can further speculated due to the upregulation of transcription factor 4 (*TCF4*, fold change 1.55, *p* < 0.0397). In addition, the increased and abundant expression of *WLS* (wntless, fold change 1.87, *p* < 0.0299) was observed on P5. 

Remarkably, osteoblasts on P5 exhibited an upregulation of antagonists of the Wnt/β-catenin pathway, such as *SOST* (sclerostin, fold change 1.57, *p* < 0.0006). Further significantly upregulated inhibitors were ICAT (inhibitor of β-catenin and TCF-4, *CTNNBIP1*; fold change 1.65, *p* < 0.0164 by weak intensity signals) as well as *SOX17* (SRY-box transcription factor 17, fold change 1.58, *p* < 0.0066), which finally antagonize the transcriptional activation of β-catenin. Another Wnt antagonist, the *SFRP4* (secreted frizzled-related protein 4, fold change −1.95, *p* < 0.0062), showed a significantly lower expression on P5 versus Ref. 

### 3.3. Topography-Dependent Protein Expression of β-Catenin Inhibitors ICAT and SOX17 

**Figure 4:**
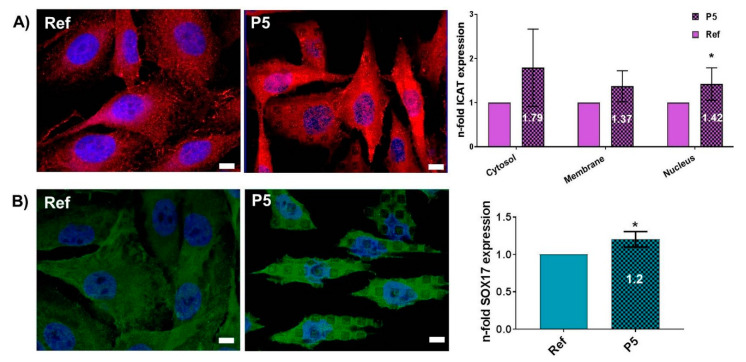
β-catenin inhibitors ICAT (inhibitor of β-catenin and TCF-4) and SOX17 (SRY-box transcription factor 17) in MG-63s on micro-pillars (P5) versus unstructured Ref after 24 h. (**A**) ICAT protein localization (red, Cy3-anti-ICAT; blue, nucleus, DAPI; LSM) and expression (ELISA). From the LSM images it is visible that on Ref the MG-63s showed a homogeneous distribution of ICAT over the entire cell. On P5 the ICAT protein appears concentrated in the nucleus (see also Figure S5). The quantification of this protein in subcellular fractions confirmed a significantly increased ICAT level in the nucleus on P5. (ELISA; Ref values normalized to 1; mean ± s.e.m.; 3 independent experiments; multiple *t*-test; * *p* < 0.05). (**B**) SOX17 protein localization (green, Alexa Fluor^®^488-anti-SOX17; blue, nucleus, DAPI) and expression (flow cytometry). Note the increased SOX17 in MG-63s on P5 observed both in immunofluorescence and flow cytometry. (FACSCalibur; Ref values normalized to 1; mean ± s.e.m. of 5 independent experiments; Mann-Whitney U-test; * *p* < 0.05). All immunofluorescence images by LSM 780; 63×, P5 image as 3D confocal z-stack overlay, scale bar: 10 μm.

The expression of β-catenin antagonists were also upregulated at protein level in MG-63s on P5 after 24 h (Figure 4). The fluorescence images revealed that on Ref the ICAT distribution appears evenly throughout the whole cell. In contrast, a higher fluorescence signal could be observed in the nucleus of cells on P5 (Figure S5) (Figure 4A left). In agreement with the fluorescence images, the quantification of ICAT protein in MG-63 subcellular fractions indicated a significantly increased nuclear and slightly higher cytosolic expression on P5 versus Ref (Figure 4A right). Also, the total protein expression of inhibitor SOX17 in MG-63s on P5 indicated a significant higher expression by both—fluorescence imaging and flow cytometry (Figure 4B).

### 3.4. Topography-Dependent Reduction of the Osteoblast-Specific Function after 96 h

**Figure 5:**
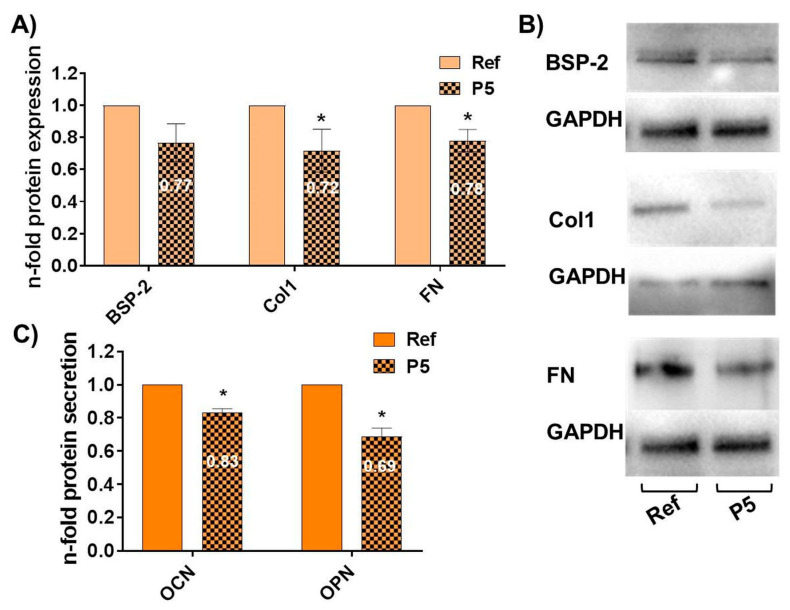
Quantification of the osteoblastic markers bone sialo protein-2 (BSP-2), collagen type I (Col1), fibronectin (FN), osteocalcin (OCN) and osteopontin (OPN) in MG-63s grown on micro-pillars (P5) or unstructured reference (Ref) for 96 h. (**A**) Immunoblotting and protein expression detection of BSP-2, Col1 and FN. (Ref values normalized to 1; mean ± s.e.m., 4 independent experiments; Mann-Whitney U test; * *p* < 0.05) (**B**) Representative Western blot bands of BSP-2 (59 kDa), Col1 (200 kDa), FN (220 kDa) and the loading control GAPDH (glyceralaldehyde 3-phosphate dehydrogenase; 37 kDa) in MG-63s on P5 compared to Ref (one out of four experiments). (**C**) Relative protein secretion of OCN and OPN by luminex assay, (Ref values normalized to 1; mean ± s.e.m., 4 independent experiments; Mann-Whitney U test, * *p* < 0.05). Osteoblast marker protein expression and secretion was impaired on micro-pillars after 96 h.

In previous studies a significant reduced expression of osteogenic markers could be observed for osteoblasts on micro-pillars after 24 h [9,12,13]. In this study the protein expression of collagen type I (Col1), fibronectin (FN) as well as protein secretion of osteocalcin (OCN) and osteopontin (OPN) displayed a significant reduction after 96 h on P5 (Figure 5).

### 3.5. Micro-Pillars Induce Oxidative Stress

**Figure 6:**
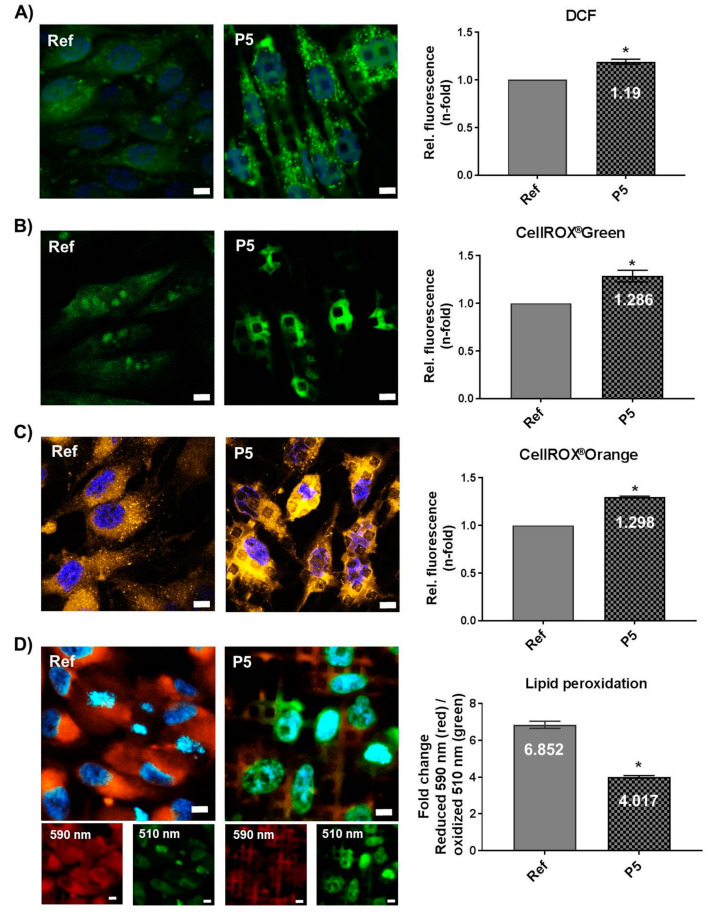
Reactive oxygen species (ROS) generation and lipid peroxidation in MG-63s on micro-pillars (P5) after 24 h; (Left) laser scanning microscopy (LSM) and (Right) Tecan reader. (**A**) ROS determination of fluorescent DCF (DCFDA cellular ROS detection assay). (**B**) Nuclear and mitochondrial determination of ROS with CellROX^®^Green reagent. (**C**) Cytosolic determination of ROS with CellROX^®^Orange reagent. (**D**) The oxidative degradation of cellular lipids was analyzed by Image-iT^®^ lipid peroxidation kit. Representative LSM images—upon oxidation fluorescence shift from 590 (red) to 510 (green) and level of lipid peroxidation as measured by the ratio of red (590 nm): green (510 nm) fluorescence. Note that micro-pillars induced significant higher ROS in the cytosol as well as in the nucleus and mitochondria. As consequence the oxidative degradation of cellular lipids occurred. (TECAN infinite M200; mean ± s.e.m., 4 independent experiments; B–C: Ref values are normalized to 1; paired *t*-test; * *p* < 0.05.). (LSM 780, Carl Zeiss; blue: nucleus, Hoechst 33324; magnification 40×, scale bar: 10 µm).

The micro-pillars (P5) induced the reactive oxygen species (ROS) production and as consequence lipid peroxidation in MG-63s (Figure 6). To investigate the ROS production after 24 h, first the DCF-DA Cellular ROS detection assay indicated elevated ROS (1.19-fold ± 0.027) in MG-63s on P5 versus Ref (Figure 6A). In addition, it can be demonstrated that P5 provoked the ROS production 1.12-fold (±0.021, * *p* < 0.05) just after 1 h cultivation (data not shown). Moreover, intracellular ROS was determined significantly in the nucleus, the mitochondria (both by CellROX^®^Green) as well as in the cytosol (CellROX^®^Orange) (Figure 6B,C). Figure 6D shows that the oxidation state of the membrane lipids for MG-63s on P5 is significantly lower on Ref, which implies an increase in lipid peroxidation due to ROS.

Antioxidative enzymes like superoxide dismutase (SOD) eliminate ROS by catalyzing reactions. The protein expression of one of these ROS-scavenging enzymes—SOD3—was significantly increased (microarray technology: fold change 1.32, *p* < 0.0345; Table S1).

### 3.6. In Silico

To complement the experimental results obtained in this study, an in silico model of the Wnt/β-catenin pathway was developed. The model allows a quantitative exploration of the complex interplay between ROS, the inhibitors and transcription factors that are differentially expressed on micro-topography (P5) compared with unstructured samples (Ref). The model and the assumptions derived from the experimental results are briefly described below.

**Figure 7:**
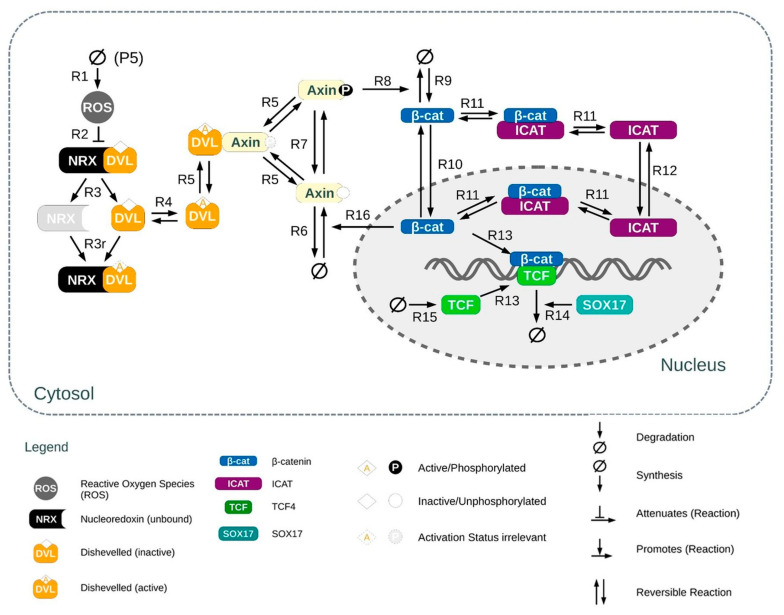
Schematic representation of the implemented model of the Wnt-independent β-catenin pathway. Arrows indicate the state change of the affected molecule, such as synthesis or degradation reactions (R1, R6, R9, R14, R15), complex formation or dissociation (R2, R3, R3r, R5, R11, R13), activation or inactivation (R4, R7), and shuttling (R10, R12). Note that double arrows represent reversible reactions/state changes. Reactions that occur more than once in the figure actually refer to one and the same reaction and have been included to increase readability. This applies in particular for complex formation, where for each molecule a separate arrow points to the final complex, while referring to the one reaction (e.g., R11: β-cat + ICAT ↔ β-cat/ICAT, β-cat (β-catenin), ICAT (inhibitor of β-catenin and TCF-4)). Arrows (or horizontal lines) that point towards another arrow indicate a promoting (or attenuating) effect of the molecule on the given reaction (R8, R14, R16). For example, SOX17 (SRY-box transcription factor 17) promotes the degradation of the β-catenin/TCF (transcription factor) complex (R14). Phosphorylated AXIN is labeled by a white P on a black circle while active, self-aggregated Dishevelled (DVL) complexes are indicated by an orange A. Please note that for some reactions the molecule’s state of activity is not relevant, e.g., the phosphorylation state of AXIN has no impact on its binding to activated Dvl (Dvl [A]). Please see the legend for a detailed description of the graphical elements used in the figure.

According to the in vitro observations, P5 induce the production of ROS in MG-63s (Figure 7). Several studies provide evidence of a cross-talk between redox and Wnt/β-catenin signaling [37,38,39,40]. Accordingly, elevated ROS levels release the redox-sensitive association between Nucleoredoxin (NRX) and DVL leading to a spontaneous increase of unbound DVL molecules, which immediately form a binding platform for AXIN, the main component of the β-catenin degradation complex [38,39]. DVL inhibits thereby β-catenin degradation, resulting in an accumulation of β-catenin in the cytosol and nucleus in a Wnt-independent manner [37]. This pathway is incorporated and validated in the in silico model presented in [28]. It forms the basis for the in silico study and is further extended.

It is assumed that there is no extracellular Wnt stimulus; i.e., Wnt proteins are neither part of the medium nor being produced and secreted by the cells. This assumption is supported by the mRNA expression profile, which provides no significant difference between Ref and P5 in the expression of Wnt ligands. Therefore, the model disregards any membrane-associated signaling components, and considers solely the intracellular signaling components.

**Figure 8:**
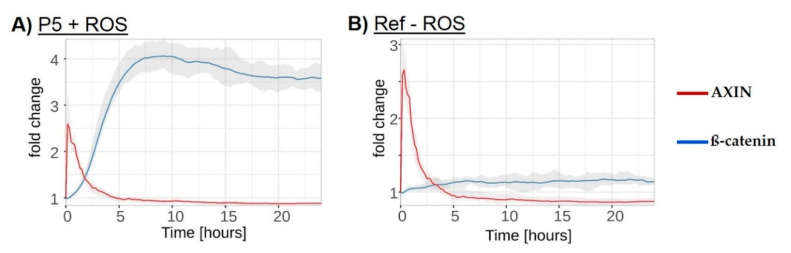
Simulation result of the fitted model showing the concentration fold change of total β-catenin (blue) and AXIN expression (red) on (**A**) P5, and (**B**) Ref. The grey area depicts the mean deviation between individual simulation runs for each time point. (**A**) The simulation of the model with higher reactive oxygen species (ROS) production, as observed on P5 (see Figure 6), results in a moderate accumulation of β-catenin, whereas the target gene expression of AXIN stays constant after a short initial increase. (**B**) The simulation of the model with no ROS production, in contrast to P5. Here was observed a small, but not significant accumulation of β-catenin, while the target gene expression of AXIN stays constant after a short initial increase (similar to P5).

Next, the intracellular components that are differentially expressed on P5, i.e., ICAT, SOX17 and TCF, were added to the model. The role of TCF was thoroughly described in the original Wnt model by Lee et al. [41] and can be seamlessly incorporated in the model with the corresponding concentration values and reaction rate constants. With regard to ICAT and SOX17 and their regulatory mechanisms, applied fitting have been used to obtain the corresponding dynamics (Figure 8). Please note, for simplicity and based on their similar function, the homologues AXIN and AXIN2 are both considered as AXIN in the model.

## 4. Discussion

The surfaces of biomaterials play a central role in the modulation of cell behavior [42,43] and in mechanotransduction [7,44,45]. Topographical features have an influence on direct transmission of force, which causes a reorganization of the cytoskeleton [31,46,47,48] and changes in cell shape to adapt to the underlying surface [44,49,50]. Furthermore, signaling pathways ultimately modulate cell proliferation and differentiation [4,5,8,10,46,51,52,53,54,55].

Wnt signaling is one of the central signaling pathway that controls cell fate specification [14,16,21,56] and to play a decisive role in bone tissue homeostasis [15,28] and in osteoblast differentiation [57,58,59,60]. Wnt signaling consists of two major pathway subgroups defined by their interaction with β-catenin: canonical or Wnt/β-catenin, and non-canonical pathways—Wnt/planar cell polarity (PCP) pathway and Wnt/calcium pathway [14,53,61,62]. Some studies indicated a specificity of Wnt signaling: the activation of both non-canonical Wnt pathways’ components may antagonize the Wnt/β-catenin pathway and vice versa [11,17,18,19,20,61,63,64]. Due to the earlier findings that actin is re-organized and Ca^2+^-release is decreased in cells on micro-pillars [9,12,13], it is assumed that both of the non-canonical signaling pathways are suppressed (Scheme 1). The question has been raised if the suppression of non-canonical Wnt pathways then could result in an activated state of Wnt/β-catenin pathway?

In the present study, the key effector of the Wnt/β-catenin pathway, the β-catenin-protein, indicated a very pronounced and effective response of MG-63s to P5 after 24 h. β-catenin protein level is strongly enhanced on P5 in cells at time periods of 1 to 24 h. β-catenin in MG-63s on the Ref appeared evenly distributed throughout the cytosol, with a stronger signal along the cell border. This can be attributed to the presence of cell-to-cell contacts due to the increased spreading area, accompanied by increased concentration of cadherin to mediate cell-cell adherence [10,16,65]. Some studies reported that N-cadherin expression is inversely related to the intracellular β-catenin level [55,57,65,66]. The micro-pillars inhibited the spreading and thus the cells were separated from each other, so that fewer cell-to-cell contacts could be established [9,12,60,67]. In addition, an increased accumulation of the β-catenin protein in the cytosol and a translocation into the nucleus was seen in MG-63s on P5. Several studies found also enhance cytosolic and nuclear β-catenin protein levels on topography [11,28,62,65,68] as marker for the activation of the Wnt/β-catenin signaling [15,16,23].

Many studies associate an activated state of the Wnt/β-catenin pathway with increased osteogenesis due to topography [11,18,45,46,54,61,69,70]. In previous work, however, a significant reduced expression of osteoblast function markers such as alkaline phosphatase (ALP), collagen type I (Col1), fibronectin (FN) and osteocalcin (OCN) could be observed on sharp-edged micro-pillars after 24 h [9,12,13]. This initial decrease in osteogenic markers was also confirmed after 96 h cultivation in the present study. 

In one study it was found that the topographical structure has an influence on the Wnt/β-catenin pathway: Schernthaner et al. [71] described in endothelial cells that structured polymers induces the translocation of β-catenin into the nucleus, while the ripple-(wave-like) nanostructure further induces the activation of specific β-catenin target genes, the initiation of β-catenin transcriptional activation was not enhanced on a wall nanostructure (pillar-like, dimension 1 µm) and was thus crucial for cell fate. Another important question in mechanotransduction arose: which effect does a defined topography exert on the β-catenin transcriptional activation? 

Transcriptome analyses by microarray technology was used to identify the full array of genes whose expression changes under topographical features. In general, the regulation of mRNA (messenger ribonucleic acid) expression in MG-63s in Wnt signaling was only observed in Wnt/β-catenin pathway components. The most important components such as Wnt, receptors or target genes were topography independent after 24 h (Table S1). Interestingly, an increase of the Frizzled-5 receptor on the protein level could be detected over time due to the micro-pillars, which was not visible on the mRNA level (Figure S6). Furthermore, the mRNA expression of β-catenin was also not affected. However, a distinct impact was detected at the protein level—β-catenin accumulation on P5—which implies a successful activation of the Wnt/β-catenin pathway. Galli et al. [46] demonstrated that topography does not directly regulate the transcription of β-catenin in C2C12 myoblast cells, but rather reduces the expression of AXIN2, which controls the degradation of β-catenin. The mRNA level exhibited a reduced expression of AXIN2 on P5 which was not significant (fold change −1.82, *p* < 0.3533). 

In the present study, six genes were identified as significantly regulated with respect to the Wnt/β-catenin pathway. The transmembrane protein Wntless displayed the highest gene expression (*WLS*) and abundancy in MG-63s on P5, which is implicated in the extracellular secretion of Wnt ligands [16,23,60]. Zhong et al. [72] could observe that the Wntless inactivation downregulated the protein level of β-catenin. In addition, the transcription factor 4 (*TCF4*) was significantly enhanced on P5. The proteins T-cell factor/lymphoid enhancer factor (TcF/LEF) family of DNA-bound transcription factors is the major partner for β-catenin that triggers specific expression of Wnt target genes [15,16,23,26]. The stabilization and nuclear accumulation of β-catenin causes TCF4 to form a complex and recruit other co-activators for gene activation, which ultimately control the cell fate [46]. Despite the upregulated *TCF4* mRNA level, the expression of target genes (like AXIN2) was topographically unchanged. Otherwise, antagonists like extracellularly secreted antagonist Sclerostin (SOST) was upregulated due to micro-topography at mRNA level. SOST binds to the LRP5/6 receptor and finally inactivates the Wnt/β-catenin pathway [23,27,56,73]. 

Two β-catenin signaling antagonists, SRY-box transcription factor 17 (*SOX17*) and inhibitor of β-catenin and TCF-4 (*ICAT*), were upregulated on P5 at mRNA level. Both antagonists are expressed and react only when β-catenin accumulates and can translocate into the nucleus, where β-catenin acts as a transcriptional co-activator [26]. ICAT binds to β-catenin in cytosol to promote the nuclear export [16]. In the nucleus, ICAT binds β-catenin to blocking the binding site (β-catenin central armadillo repeat [17,74]) for TCF4 and preventing the β-catenin/TCF transcriptional activities [14,75,76], which ultimately impairs differentiation [16,18,25,74,75]. Chen et al. [77], in their study with isolated primary Col2a1-ICAT transgenic chondrocytes, observed the inhibition of Wnt/β-catenin signaling due to ICAT overexpression, which finally delayed cell growth and maturation. Stow [78] published a review stating that the expression of the inhibitor ICAT correlates conversely with the transcriptional activity of β-catenin. Due to the higher level of the ICAT, the β-catenin is no longer able to act as a co-transcription promoter. The other negative regulator SOX17 repress the β-catenin dependent spatial and temporal LEF-1 activation [79]. Sinner et al. [26] demonstrated in their study that SOX17 interacts with both, TCF and β-catenin, and form a complex, which finally repress their transcriptional activities. Also SOX17 could be important for the regulation of the Wnt/β-catenin pathway and the manipulation of β-catenin-dependent transcriptional activation. An increased expression of both antagonists (ICAT and SOX17) in osteoblasts on P5 could also be confirmed at the protein level. As a result of the negative feedback loop, the gene expression could disturbed and thus the cell fate impaired [18,76,80]. 

The only significantly downregulated gene was secreted frizzled-related protein 4 (*SFRP4*), which interacts directly with Wnt, preventing binding to their receptor; in this way they antagonize the Wnt/β-catenin pathway [16,81]. Wang et al. [69] indicated in MG-63s on micro/nano surfaces a reduced expression of sFRP which induced enhanced nuclear amount of β-catenin. Dishevelled segment polarity protein3 (DVL3) prevents β-catenin degradation, which finally results in β-catenin accumulation in the cytosol [18,54,62]. *DVL3* showed slightly higher mRNA expression levels in MG-63s on P5 (out of cutoff criteria: fold change 1.36, * *p* < 0.0152; Table S1). In summary, the microarray gene profile provided evidence of modified Wnt/β-catenin pathway in MG-63s on sharp-edged micro-pillars. It could be demonstrate on the one hand the upregulation of factors for Wnt/β-catenin pathway activation like *WLS*, *TCF4*, *DVL*. However, on the other hand an increased mRNA expression of β-catenin inhibitors such as *SOST*, *CTNNBIP1* (ICAT) and *SOX17* suggest that the β-catenin-dependent transcriptional activity (as co-activator) is affected due to micro-topography.

Various studies demonstrated the connection between redox and Wnt/β-catenin signaling [37,38,39,40]. The regulation of β-catenin accumulation can also be achieved by the production of reactive oxygen species (ROS) in cellular differentiation processes [29,37]. In an earlier study, internalization of micro-pillars in MG-63s was demonstrated by caveolae-mediated phagocytosis, a process associated with increased ATP turnover (n-fold to Ref: 0.627 ± 0.053, * *p* < 0.05) and mitochondrial activity [13]. Furthermore, this was associated with higher ROS production and higher expression of ROS-scavenging enzyme (Catalase, n-fold to Ref: 1.151 ± 0.089, * *p* < 0.05) [13]. The present study demonstrated the increased production of ROS in cells (cytosol and nucleus) and provides new evidence that the enhanced ROS level was detectable on P5 after only 1 h cell cultivation. The generation of ROS induced finally lipid peroxidation in MG-63s on P5, a biomarker for oxidative stress. In addition, the microarray data indicated an increased expression of *SOD3* (fold change 1.32; Table S1). Hoogeboom et al. [58] identified DVL as an entry point for ROS to regulate oxidative stress. In their review they argued that under altered ROS conditions the pool of β-catenin is apparently diverted from TCF to Forkhead box-O (FOXO) transcription factors to regulate cell survival by increasing oxidative stress resistance and to support ROS degradation [58]. These antioxidative defense mechanism ultimately antagonizing the Wnt/β-catenin-dependent transcription [27,56]. In addition, Galli et al. [67] showed in their study how a SLA topography modulates the resistance of C2C12 cells to oxidative stress (stimulation with H_2_O_2_) by inhibiting TCF-mediated transcription for activation of the FOXO/β-catenin pathway. The mRNA expression level of FOXO displayed only a very slight increase due to the topography (like *FOXO1*, fold change 1.04; *FOXO6*, fold change 1.22; Table S1). One further possibility for the β-catenin accumulation in MG-63s on P5 without β-catenin-dependent transcriptional activation could be the diversion of β-catenin for the FOXO/β-catenin pathway as a defense mechanism. 

### 4.1. Redox-Dependent β-Catenin Accumulation

**Figure 9:**
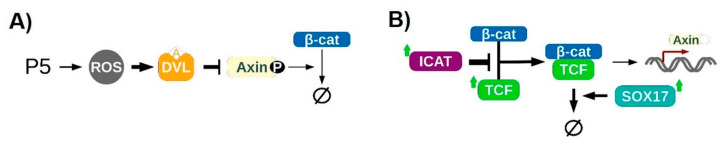
Simplified representation of (**A**) the signal pathway from reactive oxygen species (ROS) production (P5 induced) to eventual accumulation of β-catenin. Briefly, MG-63s respond to P5 with increased production of ROS, which inhibit AXIN-mediated degradation of β-catenin through Dishevelled (DVL). The width of the lines is proportional to the reaction kinetics (flux) through each reaction compared to Ref. Note that β-catenin accumulation typically induces increased AXIN synthesis. However, this is attenuated by the specific expression pattern, as shown in (**B**) the transcriptional regulation of β-catenin target gene expression on P5. A briefly elevated gene expression of T-cell specific transcription factor 4 (*TCF4*), inhibitor of β-catenin and TCF-4 (ICAT) and SRY-box transcription factor 17 (*SOX17*) leads to attenuated formation of the TCF/β-catenin complex (by ICAT) and proteosomal degradation of the β-catenin complex (by SOX17). The β-catenin target gene expression is thereby kept level despite its intracellular aggregation. The width of the lines is proportional to the reaction kinetics (flux) through each reaction compared to Ref.

While the connection between these experimental measurements is not immediately obvious, it becomes apparent when considering the in silico model. As shown in [28,37], elevated ROS levels activate DVL in a redox-dependent and Wnt-independent manner. In its inactive state, DVL is primarily bound to Nucleoredoxin (NRX), a specific member of the thioredoxin-related redox-regulating protein family. The redox-sensitive association between NRX and DVL, however, is released upon elevated intracellular ROS levels [37]. This leads to a spontaneous increase of unbound DVL molecules. Interestingly, DVL proteins are capable of forming self-aggregates if their local concentration passes a certain threshold. DVL self-aggregates present a high-affinity binding platform for AXIN [29,37,82]. Notably, due to its low copy number, AXIN is a crucial component of the β-catenin degradation complex [16,24]. Its binding to DVL leads to an inhibition of β-catenin degradation, resulting in an accumulation of β-catenin in the cytosol and nucleus (Figure 9A). 

### 4.2. Regulation of the Transcriptional Activity of β-Catenin

Typically, β-catenin accumulation in the nucleus leads to an elevated expression of WNT/β-catenin target genes, such as *Myc*, *c-Jun* and *AXIN2*. Notably, *AXIN2* has a similar function as its homolog AXIN and induces the degradation of β-catenin. This means that nuclear β-catenin accumulation usually induces a negative feedback by increasing the expression of its own inhibitor (Figure 9B). Surprisingly, the expression of β-catenin target genes is not increased on P5, despite the significant aggregation of β-catenin in the nucleus. This leads to two major questions that the model aims to answer: how do the inhibitors of the TCF/β-catenin complex such ICAT and SOX17 interact (quantitatively) with β-catenin to inhibit its transcriptional function; and at the same time, what keeps β-catenin aggregation in balance without the elevated transcription of its inhibitor AXIN2? To answer these questions, the role of the differentially expressed intracellular components in the model was analyzed, i.e., ICAT, SOX17 and TCF. Accordingly, ICAT is located in the cytosol as well as in the nucleus. Its inhibiting function is based on the competitive (reversible) binding of β-catenin [14,16]. SOX17, on the other hand, only acts inside the nucleus, directly binds to the TCF/β-catenin complex and, in contrast to ICAT, induces the degradation of the TCF/β-catenin complex [26]. Note that this degradation is independent of the cytosolic destruction complex, hence independent of AXIN and GSK-3β. The difference between their localization and the inhibiting function of ICAT and SOX17 has major implications on their impact on β-catenin aggregation and transcriptional activity. The competitive binding of ICAT to β-catenin, for instance, reduces the amount of β-catenin available for its binding to TCF, hence attenuating the transcription; at the same time, it allows an accumulation of β-catenin because it does not induce the degradation of β-catenin. However, for a sustained effect (i.e., attenuating the transcriptional activity of β-catenin) the amount of ICAT needs to match the number of β-catenin. Since β-catenin is highly abundant in the cell, a very high quantity of ICAT would be required to attenuate target gene expression. Here SOX17 acts much more efficiently: since SOX17 targets the entire TCF/β-catenin for proteasomal degradation, both β-catenin and TCF are degraded. While β-catenin is highly abundant, TCF is typically available in a much lower quantity. As a result, even a relatively small increase in the quantity of SOX17 can significantly lower the transcription and expression of β-catenin target genes. However, such an increase of SOX17 needs to be accompanied by a similarly increased expression rate of TCF; otherwise, the system reaches a point where no TCF is available and target gene expression is completely inhibited. 

### 4.3. Assembling the Entire View

Relating the insights gained from the model to the in vitro data obtained in MG-63s after cultivation on P5 provides a comprehensive picture of how, in contrast to Ref, β-catenin upregulation and translocation with constant/unchanged transcriptional activity is achieved. At first, the increased and constant production of ROS on P5 (Figure 9) induces a redox-dependent accumulation of β-catenin. To inhibit β-catenin target gene activation, such as *Myc*, *c-Jun* or *AXIN2*, MG-63s express the specific inhibitors ICAT and *SOX17* (with similar fold changes; Table 1) that act concurrently. While ICAT prevents a major fraction of β-catenin from binding to TCF, *SOX17* targets the remaining, successfully established TCF/β-catenin complexes for proteasomal degradation. However, the cells have to encounter the *SOX17*-mediated degradation of TCF to prevent a decrease or total loss of transcriptional activity. Notably, the expression of β-catenin target genes is unchanged between P5 and Ref. Therefore, *TCF4* expression is also elevated with a similar fold change as *SOX17*. Based on this coordinated expression of regulatory elements of the Wnt/β-catenin pathway, MG-63s are able to cope with the ROS-induced accumulation of β-catenin and suppress an unintended target gene expression. 

### 4.4. In Summary

**Figure 10:**
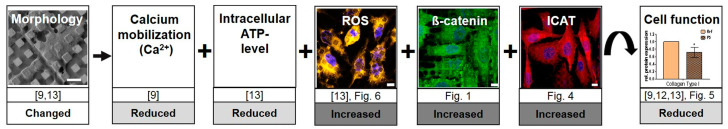
Summary of the complex intracellular responses of MG-63s on micro-pillars which all contribute to reduced cell functions. The sharp-edged micro-pillars (P5) changed the cell morphology (FE-SEM Supra25, Carl Zeiss; Figure S1) [9,13] and hampered the calcium mobilization [9]. Furthermore, a reduced adenosine triphosphate (ATP)-content [13] as well as an increased reactive oxygen species (ROS) production due to cellular stress could be observed (Figure 6) [13]. The protein-level of β-catenin as well as the translocation into the nucleus was increased (Figure 1), but antagonists such as ICAT (inhibitor of β-catenin and TCF-4) (Figure 4) finally lead to an inhibited transcriptional activation. As a consequence, osteoblasts were reduced in their extracellular matrix-synthesis like collagen type I (Figure 5) [9,12,13]. (Scale bars 10 µm).

Defined sharp-edged micro-pillars caused a change in cell morphology (Figure S1), reduced the calcium (Ca^2+^) mobilization and induced a cellular stress reaction with low adenosine triphosphate (ATP) levels and increased ROS production [13]. In the present study it was shown that the Wnt/β-catenin pathway was regulated by the micro-topography—β-catenin accumulation and translocation into the nucleus (Figure 10). The ultimately impairment of osteogenesis [9,12,13] could be caused by an inhibition of the TCF/β-catenin transcriptional activity due the antagonists ICAT and SOX17 or via other pathways involved in the regulation of β-catenin as co-activator (FOXO/β-catenin signaling pathway) [14,20,68]. However, possible mechanisms how the cell fate is then influenced by the regulation of transcriptional co-activity, was revealed by the in silico study, and confirmed by the increased expression of the inhibitors ICAT and SOX17 as well as TCF4. In addition, the higher cellular oxidative stress on P5 initiates the accumulation of β-catenin and subsequent redirection into other signaling pathways, such as the defense mechanism against ROS.

Other antagonistic effects on Wnt signaling in MG-63 osteoblasts, such as hypoxia [83], treatment with inhibitors of Wnt/β-catenin-mediated transcription PRI-724 [84] and Resveratrol [85] could found in other experiments. Upcoming studies are needed to understand the downstream mechanism of molecular events that control cell behavior induced by micro-topography. In this context, topography properties such as dimension, shape, or smoothness should be modulated, and then correlated with their effects on the Wnt/β-catenin pathway. Cell-stimulatory or inhibitory effects of topographical surface properties of a biomaterial can be studied further in silico to better understand cell behavior. It is important to elucidate possible molecular mechanisms for the design of metal biomaterials in regenerative medicine with improved acceptance and osseointegration.

## Figures and Tables

**Table 1:** Overview of significantly expressed genes related to Wnt/β-catenin pathway in MG-63s on micro-pillars (P5) compared with unstructured reference (Ref) (positive z-score value means a gene expression increase in MG-63s on P5 (red) and a negative z-score value means a lower gene expression on P5 versus Ref (green); microarray technology, triplicates).

Gene Symbol	Description	P5 Avg (log2)	Ref Avg (log2)	Fold Change (P5 versus Ref)	*p*-Value
*WLS*	Wntless, Wnt ligand secretion mediator	12.8	11.89	1.87	0.0299
*CTNNBIP1*	ICAT, catenin beta interacting protein 1	8.17	7.45	1.65	0.0006
*SOX17*	SRY-box transcription factor 17	4.02	3.36	1.58	0.0066
*SOST*	sclerostin	3.74	3.08	1.57	0.0164
*TCF4*	transcription factor 4	11.56	10.93	1.55	0.0397
*SFRP4*	secreted frizzled-related protein 4	4.26	5.23	−1.95	0.0062

## Supplementary Materials

The following supplementary materials are available online at https://www.mdpi.com/2073-4409/9/8/1784/s1, Figure S1: Cell phenotype, Figure S2: Proof of subcellular fractionation and western blot, Figure S3: Proof of ROS generation, Figure S4: Source code of the Wnt model in ML-Rules, Figure S5: ICAT expression and localization, Figure S6: Frizzled expression, Table S1: Overview of genes, Table S2: Parameter values of the Wnt model.
