# Supplementary material for: ROS Dependent Wnt/β-Catenin Pathway and Its Regulation on Defined Micro-Pillars—A Combined In Vitro and In Silico Study
Source: Cells. 2020 Jul 27;9(8):1784. doi: 10.3390/cells9081784 (PMC7464713; doi:10.3390/cells9081784)
Supplement: Supplementary file 1 [file cells-09-01784-s001.zip › Supplementary material_Staehlke/Figure S1_Cell phenotype.pdf]

Supplementary material Figure S1

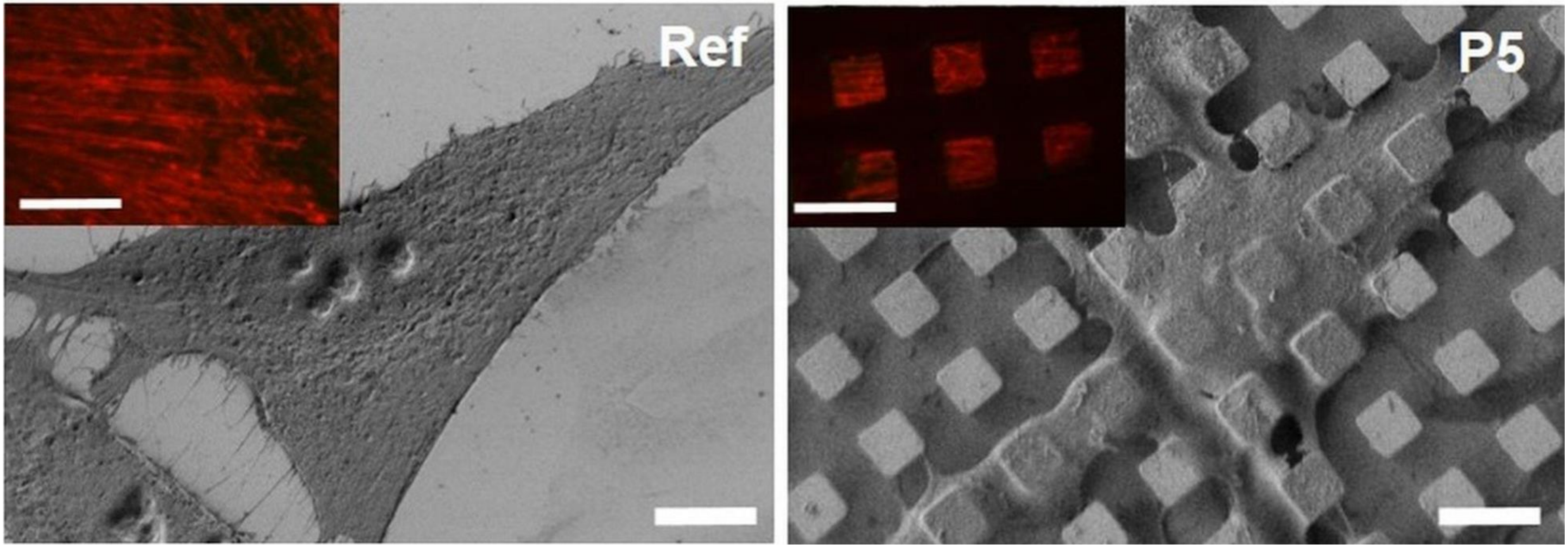

Figure S1. Cell phenotype of MG-63s on micro-pillars (P5, right) compared to unstructured reference (Ref, left) after 24 h. (Cell morphology, scanning electron microscopy, FE-SEM Supra25, Carl Zeiss; Inserts: actin cytoskeleton of phalloidin TRITC stained cells, LSM 780, Carl Zeiss, 63x oil-immersion objective; P5 immunofluorescent image is 3D confocal z-stack overlay; scale bars 10 µm). Note that cells on P5 adhered on the top of the pillars and the actin cytoskeleton was reorganized in short fibers [Staehlke et al., Moerke et al.].

Staehlke, S.; Koertge, A.; Nebe, B. Intracellular calcium dynamics dependent on defined microtopographical features of titanium. Biomaterials. 2015, 46, 48-57. https://doi.org/ 0.1016/j.biomaterials.2014.12.016.

Moerke, C.; Mueller, P.; Nebe, B. Attempted caveolae-mediated phagocytosis of surface-fixed micro-pillars by human osteoblasts. Biomaterials. 2016, 76. 102-114. https://doi.org/10.1016/j.biomaterials.2015.10.030.
