# Supplementary material for: ROS Dependent Wnt/β-Catenin Pathway and Its Regulation on Defined Micro-Pillars—A Combined In Vitro and In Silico Study
Source: Cells. 2020 Jul 27;9(8):1784. doi: 10.3390/cells9081784 (PMC7464713; doi:10.3390/cells9081784)
Supplement: Supplementary file 1 [file cells-09-01784-s001.zip › Supplementary material_Staehlke/Figure S2_Proof of subcellular fractionation and western blot.pdf]

Supplementary material Figure S2

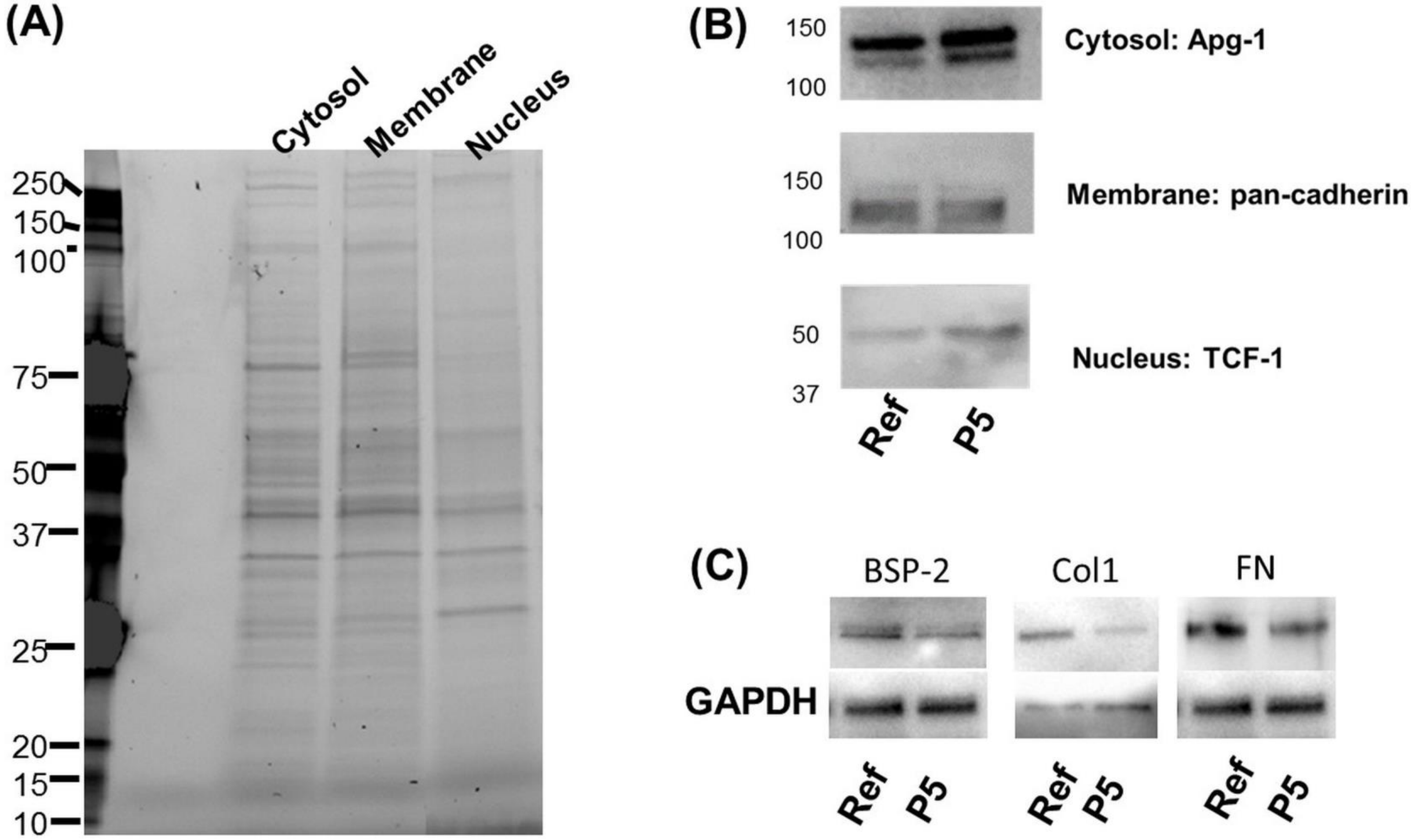

Figure S2. Endogenous control of subcellular fractionation (2.2; A/B) and Western Blot (2.6; C). (A) Stepwise extractions clearly showed distinct protein patterns of different subcellular fractions in the stain free gel with ChemiDoc™ MP imager (Bio-Rad). (B) Assign selected marker proteins were detected in the corresponding subcellular fraction by immunoblotting: primary antibody (1:1,000; mouse): Cytosol – Apg1 (120 D-12; Heat Shock Protein Family A; Santa Cruz Biotechnology, Inc.), Membrane – pan-cadherin (E-11; Santa Cruz); Nucleus – TCF-1 (T cell factor-1; C-5; Santa Cruz); secondary antibody horseradish peroxidase (HRP)-conjugated monoclonal anti mouse IgG (Dako Denmark A/S: 1:10,000). (C) GAPDH (glyceralaldehyde 3-phosphate dehydrogenase, A-3; Santa Cruz; 1:1000; mouse) was used as general loading control. (unstructured reference (Ref) vs. micro-pillars (P5)).
