# Supplementary material for: ROS Dependent Wnt/β-Catenin Pathway and Its Regulation on Defined Micro-Pillars—A Combined In Vitro and In Silico Study
Source: Cells. 2020 Jul 27;9(8):1784. doi: 10.3390/cells9081784 (PMC7464713; doi:10.3390/cells9081784)
Supplement: Supplementary file 1 [file cells-09-01784-s001.zip › Supplementary material_Staehlke/Figure S3_Proof of ROS generation.pdf]

# Supplementary material Figure S3

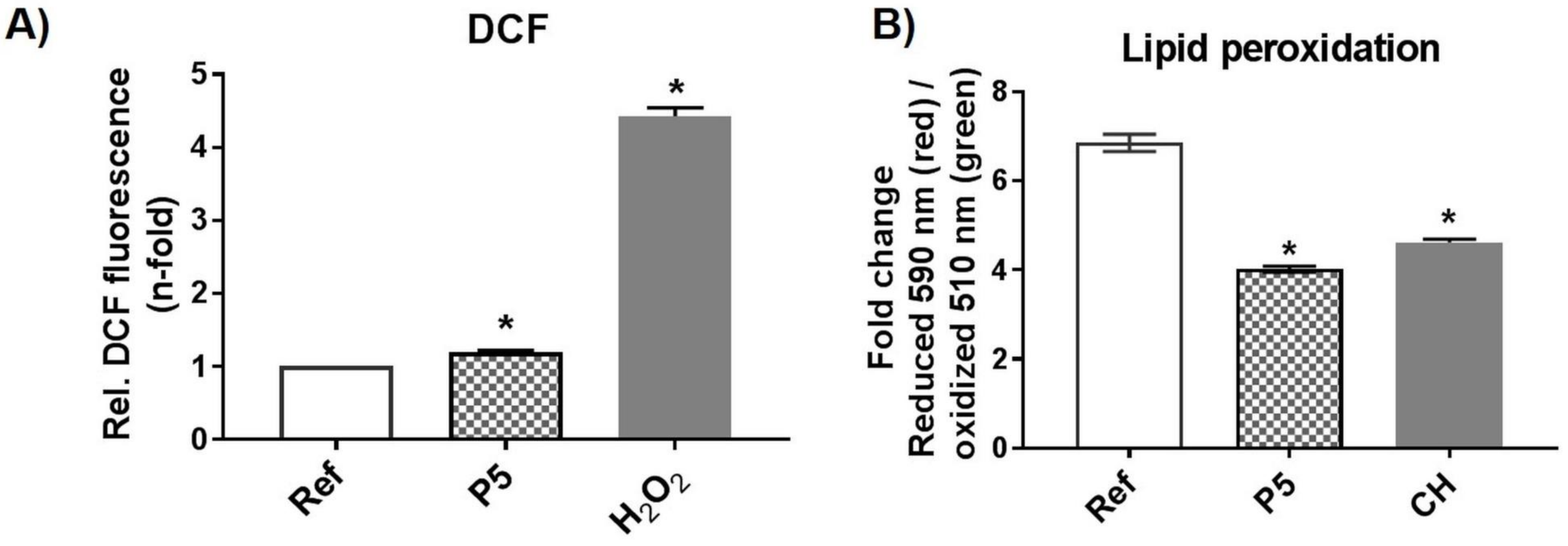


**Figure S3.** Proof of reactive oxygen species (ROS) generation and lipid peroxidation in MG-63s after 24 h. (A) An positive control for the DCFDA cellular ROS detection assay kit was carried out by exposing MG-63 osteoblasts with $H_2O_2$ (hydrogen peroxide, 2 mM) by incubation. Note the high ROS generation under positive control. (B) As positive control for the Image-iT® Peroxidation Kit, cells were treated with or 100 µM CH (cumene hydroperoxide) for 2 h at 37 °C. Note that the treatment with CH increased the proportion of oxidized membrane lipids, resulting in a significant decrease of the fluorescence intensity ratio. (TECAN infinite M200; mean + s.e.m., 4 independent experiments; A: Ref values are normalized to 1; One-way ANOVA post hoc uncorrected Fisher's LSD; *$p < 0.05$., unstructured reference (Ref), micro-pillars (P5)).
