# Supplementary material for: ROS Dependent Wnt/β-Catenin Pathway and Its Regulation on Defined Micro-Pillars—A Combined In Vitro and In Silico Study
Source: Cells. 2020 Jul 27;9(8):1784. doi: 10.3390/cells9081784 (PMC7464713; doi:10.3390/cells9081784)
Supplement: Supplementary file 1 [file cells-09-01784-s001.zip › Supplementary material_Staehlke/Figure S4_Source code of the Wnt model in ML Rules.pdf]

```
// ++++++++++++++++++++++++++++++++++++
// ++++++ initial species counts ++++++
// ++++++++++++++++++++++++++++++++++++

// ** beta-catenin signalling **
nbetacyt: 12989;
nbetanuc: 5282;
nAxin:   252;
nAxinP:  219;

// ** Ros-Dvl signalling **
nRos: 0;
nDvl: 855;
nNrx: 18;
nDvlNrx: 166200;

// ** regulatory factors **
nICAT: 1200;
nSox: 100;
nTCF: 7714;

//in common
nCells:  1;

// ++++++++++++++++++++++++++++++++++++++
// +++++ reaction rate coefficients +++++
// ++++++++++++++++++++++++++++++++++++++

// ** beta-catenin signalling **

kApA:        0.03;    // k7r Basal dephosphorylation of AxinP
kAAp:        0.03;    // k7  Phosphorylation of Axin
kApdeg:      4.48E-3;    // k6  Degradation of phosphorylated Axin
kAdeg:       4.48E-3;    // k6  Degradation of unphosphorylated Axin
kAsyn:       4E-4;    // k16 Axin synthesis (beta-catenin mediated)

kbetasyn:    600;     // k9  beta-catenin synthesis
kbetadeg_act: 2.1E-4;    // k8  axin-induced degradation of beta-catenin
kbetadeg:    1.13E-4;    // k9r basal degradation of beta-catenin
kbetain:     0.0549;     // k10 beta-catenin shuttling into nucleus
kbetaout:    0.135;   // k10r beta-catenin shuttling out of nucleus

// ** Ros-Dvl Signalling **
kRosSyn:   100;    // k1   ROS synthesis

// Nrx
kNrxRos:   5E2;    // k2_h Oxidation of Nrx by Ros
kNrxNo:    2E-2;   // k3r_h Reduction of Nrx

// Dvl
kDvlSponAgg: 5E-04;      // k4  spontaneous aggregation of Dvl
kDvldisAgg: 0.5;   // k4r basal disscoiation of Dvl aggregates

// Dvl-Nrx
kDvlNrxBind:  22.5;      // k3r Binding of Nrx and Dvl
kDvlNrxUnbind: 2.3E-2;   // k3  Basal unbinding of Nrx and Dvl
kDvlNrxRos: 3.2E2;    // k2  Unbinding of Nrx and Dvl complex by ROS

// Dvl-Axin
kDvlAxinBind: 0.075;    // k5  Binding of (activated) DVL and Axin
kDvlAxinUnbind: 6.8E-2;  // k5r Unbinding of Dvl/Axin complex

// regulatory factors
kICATsyn: 250;     // k_h2 Increasing ICAT concentration
ka_IcatBcat: 0.1;     // k11 Binding of ICAT and beta-catenin
kd_IcatBcat: 0.032;   // k11r Unbinding of ICAT and beta-catenin complex

ka_TcfBcat: 0.00196;     // k13 Binding of TCF and beta-catenin
kd_TcfBcat: 0.0141;   // k13r Unbinding of TCF and beta-catenin
kTcfSyn: 0.029;    // k15 Synthesis of TCF

kSox: 2.3e-4;      // k14 degradation of TCF/beta-catenin complex


// ++++++++++++++++++++++++++++++++++++++++++++++++++++++
// +++++ species definitions (number of attributes) +++++
// ++++++++++++++++++++++++++++++++++++++++++++++++++++++

Cell()[];
Nuc()[];
```

```
// species definitions (number of attributes)
Axin(string); // phosphorylation state
Bcat(string); // activation state
Dvl(string); // aggregation state
Nrx(string); // oxidation state
DvlNrx();
Ros(string); // activated

DvlAxin(string); // phosphorylation state Axin

Dummy();
ICAT();
Sox17();
TCF();

P();

// ++++++++++++++++++++++++++++
// +++++ initial solution +++++
// ++++++++++++++++++++++++++++
//merging from M1 and M2
>>INIT[
      (nCells) Cell[
       0 P +
       (nDvl) Dvl(’i’) +
       0 Dvl(’a’) +
       (nNrx) Nrx(’nO’) +
       0 Nrx(’O’) +
       (nDvlNrx) DvlNrx +
       0 DvlAxin(’u’) +
       0 DvlAxin(’p’) +
       (nRos) Ros(’i’) +
       0 Ros(’a’) +
       (nbetacyt) Bcat(’a’) +
       0 Bcat(’i’) +
       0 ICAT +
       nAxin Axin(’u’) +
       nAxinP Axin(’p’) +
       Nuc()[(nbetanuc) Bcat(’a’) +
      0 Bcat(’i’) +
      0 Bcat(’c’) +
         (nICAT) ICAT +
      (nSox) Sox17 +
      (nTCF) TCF
       ] +
    Dummy()
    ]
];


// +++++++++++++++++++++++++++++++
// +++++ reaction rules ++++++++++
// +++++++++++++++++++++++++++++++

// (H1) - constant production of counting Species
Cell[s?] -> Cell[P + s?] @ 1;

// **** Ros-Dvl Signalling ****

// (R1) Ros Synthesis
Cell[P:p + s?] -> Cell[Ros(’a’) + P + s?] @ kRosSyn*(1+(#p/1000));

// (R2) Forced Unbinding of Dvl from Nrx by Ros
Cell[DvlNrx:dn + Ros(’a’):r + s?] -> Cell[Dvl(’i’) + Nrx(’O’) + s?] @kDvlNrxRos*#dn*#r;

// (R2_h) Oxidation of Nrx by Ros
Cell[Nrx(’nO’):n + Ros(’a’):r + s?] -> Cell[Nrx(’O’) + s?] @ kNrxRos*#n*#r;

// (R3) Basal unbinding of Dvl from Nrx
DvlNrx:dn -> Dvl(’i’) + Nrx(’nO’) @ kDvlNrxUnbind*#dn;

// (R3r) Binding of Dvl by Nrx
Cell[Dvl(’i’):dvl + Nrx(’nO’):n + s?] -> Cell[DvlNrx + s?] @ kDvlNrxBind*#dvl*#n;

// (R3r_h) Reduction of Nrx
Nrx(’O’):n -> Nrx(’nO’) @ kNrxNo*#n;

// (R3r/R4) Forced Disaggregation of Dvl by un-oxidized Nrx
Cell[Dvl(’a’):dvl + Nrx(’nO’):n + s?] -> Cell[DvlNrx + s?] @kDvlNrxBind*#dvl*#n;
```

```
// (R4) Activation (by e.g. aggregation) of Dvl
Dvl('i'):dvl -> Dvl('a') @ kDvlSponAgg*#dvl;

// (R4r) Dynamic deactivation (e.g. by disaggregation) of Dvl
Dvl('a'):dvl -> Dvl('i') @ kDvldisAgg*#dvl;

// **** Axin Dvl signalling ****

// (R5) Axin binding by activated Dvl
Cell[Dvl('a'):dvl + Axin(phos):a + s?] -> Cell[DvlAxin(phos) + s?]@kDvlAxinBind*#dvl*#a;

// (R5r) Axin Dvl unbinding
Cell[DvlAxin(phos):da + s?] -> Cell[Dvl('a') + Axin(phos) + s?] @kDvlAxinUnbind*#da;

// (R6) Axin degradation
Axin(x):a -> @ kApdeg*#a;

// (R7r) Basal AxinP dephosphorylation
Axin('p'):a -> Axin('u') @ kApA*#a;

// (R7) Axin phosphorylation
Axin('u'):a -> Axin('p') @ kAAp*#a;

// **** Beta-catenin signalling ****

// (R8) Activated beta-catenin degradation
Cell[Axin('p'):a + Bcat('a'):b + s?]:c -> Cell[Axin('p') + s?] @ #c*((kbetadeg_act*#a*#b));
Cell[Axin('p'):a + Bcat('i'):b + s?]:c -> Cell[Axin('p') + s?] @ #c*((kbetadeg_act*#a*#b));
Cell[Axin('p'):a + Bcat('c'):b + s?]:c -> Cell[Axin('p') + s?] @ #c*((kbetadeg_act*#a*#b));

// (R9) Beta-catenin synthesis
Cell[s?]:c -> Cell[Bcat('a') + s?] @ #c*kbetasyn;

// (R9r) Basal beta-catenin degradation
Bcat(x):b -> @ kbetadeg*#b;

// (R10) Beta-catenin shuttling into the nucleus
Bcat('a'):b + Nuc[s?] -> Nuc[Bcat('a') + s?] @ kbetain*#b;
Bcat('i'):b + Nuc[s?] -> Nuc[Bcat('i') + s?] @ kbetain*#b;

// (R10r) Beta-catenin shuttling out of the nucleus
Nuc[Bcat('a'):b + s?] -> Bcat('a') + Nuc[s?] @ kbetaout*#b;
Nuc[Bcat('i'):b + s?] -> Bcat('i') + Nuc[s?] @ kbetaout*#b;

// *************************
// ICAT and SOX17 signaling
// *************************

// (H2) increasing ICAT concentration
Cell[s?] -> Cell[ICAT + s?] @ kICATsyn;

// (R11) ICAT binding beta-catenin
ICAT:ic + Bcat('a'):b -> Bcat('i') @ ka_IcatBcat * #ic * #b;

// (R11r) unbinding of ICAT and beta-catenin
Bcat('i'):b -> ICAT + Bcat('a') @ kd_IcatBcat*#b;

// (R12) ICAT shuttling into the nucleus
ICAT:i + Nuc[s?] -> Nuc[ICAT + s?] @ kbetain*#i;

// (R12r) ICAT shuttling out of the nucleus
Nuc[ICAT:i + s?] -> ICAT + Nuc[s?] @ kbetaout*#i;

// (R13) TCF binding beta-catenin
Nuc[Bcat('a'):b + TCF:c + s?] -> Nuc[Bcat('c') + s?] @ ka_TcfBcat * #b*#c;

// (R13r) unbinding of TCF and beta-catenin
Nuc[Bcat('c'):b + s?] -> Nuc[Bcat('a') + TCF + s?] @ kd_TcfBcat * #b;

// (R14) Degradation of TCF/beta-catenin complex
Nuc[Sox17:s + Bcat('c'):b + sn?] -> Nuc[Sox17 + sn?] @ kSox*#s*#b;

// (R15) TCF synthesis
Nuc[sn?] -> Nuc[TCF + sn?] @ kTcfSyn;

// (R16) Axin synthesis
Nuc[Bcat('c'):b + s?] -> Nuc[Bcat('c') + s?] + Axin('u') + Dummy @ kAsyn*#b;
```

Listing 1: Source Code of Wnt pathway model in ML-Rules.
