# Supplementary material for: ROS Dependent Wnt/β-Catenin Pathway and Its Regulation on Defined Micro-Pillars—A Combined In Vitro and In Silico Study
Source: Cells. 2020 Jul 27;9(8):1784. doi: 10.3390/cells9081784 (PMC7464713; doi:10.3390/cells9081784)
Supplement: Supplementary file 1 [file cells-09-01784-s001.zip › Supplementary material_Staehlke/Figure S5_Expression and localization of ICAT.pdf]

Supplementary material Figure S5

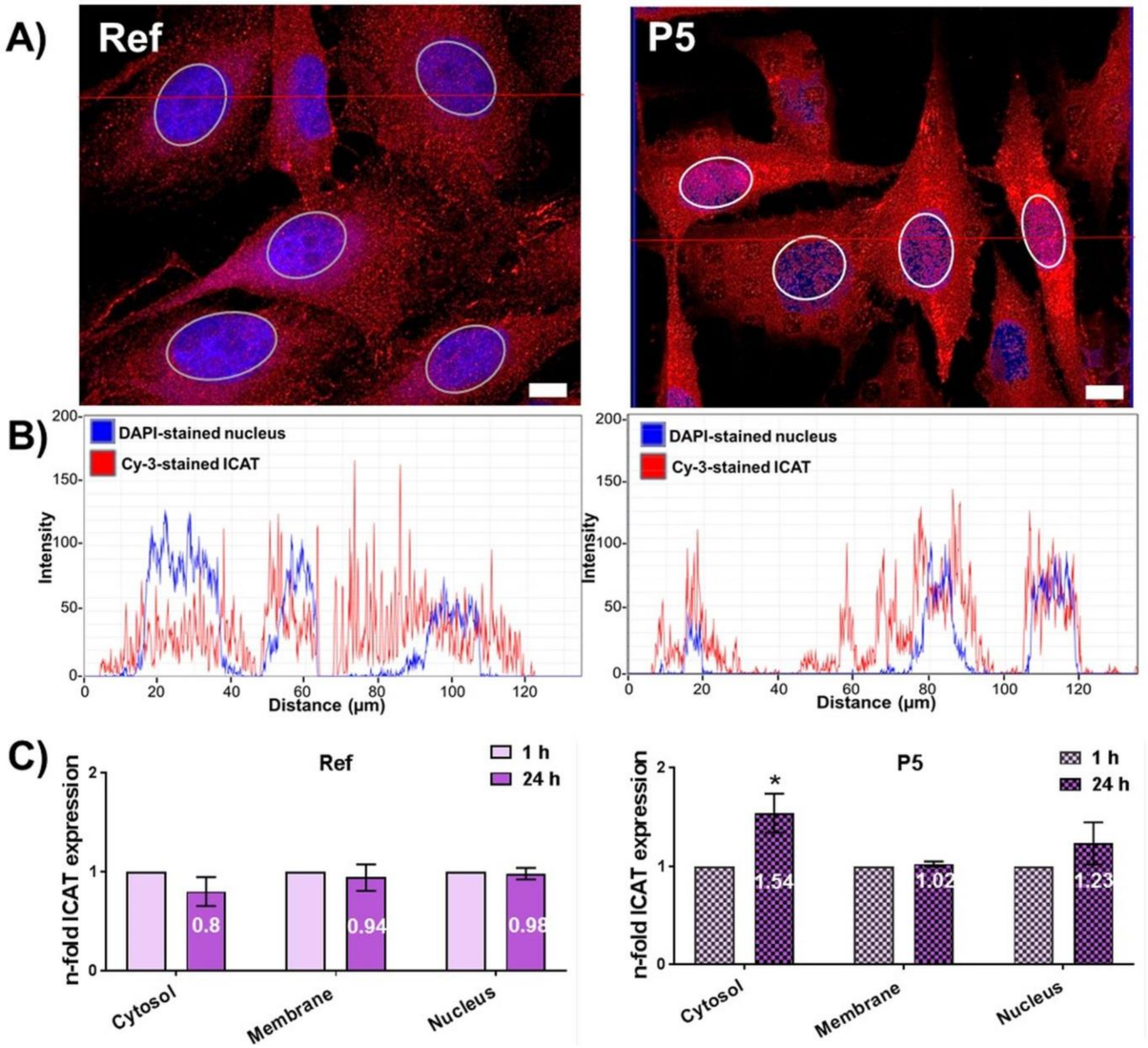

Figure S5. Expression and localization of the inhibitor of β-catenin and TCF-4 (ICAT) in MG-63s on micro-pillars (P5, right) vs. unstructured reference (Ref, left). (A) Immunofluorescence of ICAT in MG-63s after 24 h (red: Cy3 (Indocarbocyanine)-anti-ICAT, blue: nucleus, DAPI (4′,6-diamidino-2-phenylindole); LSM 780, Carl Zeiss; magnification 63x, P5 immunofluorescent image is 3D confocal z-stack overlay, scale bar: 10 µm). Note that on Ref, the MG-63s showed a homogeneous distribution of ICAT over the entire cell. In contrast, the concentration of ICAT in the nucleus of cells on P5 appears increased. (B) Profile of fluorescence intensity recorded at specific region across the fluorescence images (red line in A). Note on P5 the ICAT profile (red) is detectable in the cytosol as well as in the nucleus (blue) with the same intensity. (Zeiss, ZEN function “Profile”; Pinhole section: 0.7 µm). (C) Time dependent ICAT protein expression in MG-63 subcellular fractions on P5 (1 h vs. 24 h). At the time points 1 h and 24 h an increase was detectable on P5 in the cytosol and only marginally in the nucleus, whereas on Ref the expression was time-independent. (Competitive ELISA; 1 h values normalized to 1; mean ± s.e.m., at least 3 independent experiments; multiple t-test; *$p<0.05$).
