# Supplementary material for: ROS Dependent Wnt/β-Catenin Pathway and Its Regulation on Defined Micro-Pillars—A Combined In Vitro and In Silico Study
Source: Cells. 2020 Jul 27;9(8):1784. doi: 10.3390/cells9081784 (PMC7464713; doi:10.3390/cells9081784)
Supplement: Supplementary file 1 [file cells-09-01784-s001.zip › Supplementary material_Staehlke/Figure S6_Frizzled expression.pdf]

Supplementary material Figure S6

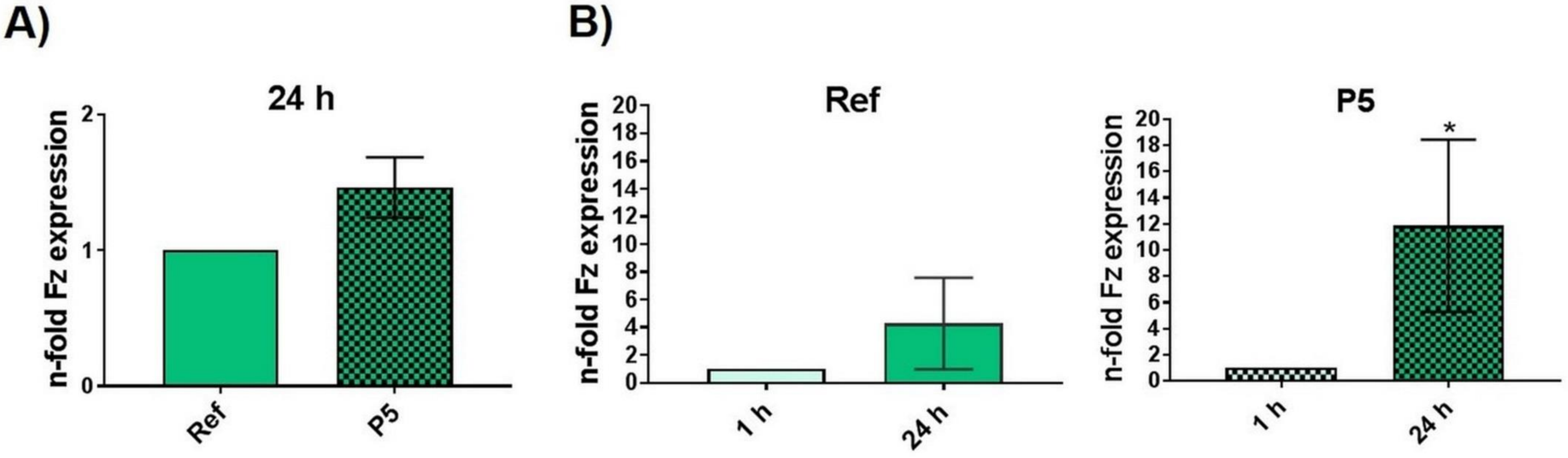

Figure S6. Flow cytometric analysis of Frizzled-5 (Fz) protein expression (primary antibody: anti-Frizzled 5, Abcam; 1:50, 30 min; secondary antibody: Alexa Fluor® 488-labeled goat anti-rabbit, Life technologies, 1:200) (FACSCalibur, BD Biosciences). (A) Fz protein expression in MG-63s on unstructured reference (Ref) vs. micro-pillars (P5) after 24 h. Note that Fz was slightly but not significantly more highly expressed on P5. (Ref values normalized to 1; mean ± s.e.m. of 5 independent experiments; Mann-Whitney U test; n.s.). (B) Time-dependent Fz receptor expression after 1 h and 24 h on Ref and P5. Note the increased expression of Fz in MG-63s on P5 after 24 h. (1 h-values normalized to 1; mean ± s.e.m., 5 independent experiments; Mann Whitney U test; $^{*}p<0.05$).
